# Mitochondrial Genome Variants and Nuclear Mitochondrial DNA Segments in 7331 Individuals from NyuWa and 1KGP

**DOI:** 10.1093/gpbjnl/qzaf098

**Published:** 2025-11-05

**Authors:** Yuanxin Wang, Jiajia Wang, Yanyan Li, Peng Zhang, Zhonglong Wang, Shuai Liu, Yiwei Niu, Yirong Shi, Sijia Zhang, Tingrui Song, Tao Xu, Shunmin He

**Affiliations:** State Key Laboratory of Epigenetic Regulation and Intervention, Institute of Biophysics, Chinese Academy of Sciences, Beijing 100101, China; College of Life Sciences, University of Chinese Academy of Sciences, Beijing 100049, China; State Key Laboratory of Epigenetic Regulation and Intervention, Institute of Biophysics, Chinese Academy of Sciences, Beijing 100101, China; State Key Laboratory of Epigenetic Regulation and Intervention, Institute of Biophysics, Chinese Academy of Sciences, Beijing 100101, China; State Key Laboratory of Epigenetic Regulation and Intervention, Institute of Biophysics, Chinese Academy of Sciences, Beijing 100101, China; State Key Laboratory of Epigenetic Regulation and Intervention, Institute of Biophysics, Chinese Academy of Sciences, Beijing 100101, China; College of Life Sciences, University of Chinese Academy of Sciences, Beijing 100049, China; State Key Laboratory of Epigenetic Regulation and Intervention, Institute of Biophysics, Chinese Academy of Sciences, Beijing 100101, China; State Key Laboratory of Epigenetic Regulation and Intervention, Institute of Biophysics, Chinese Academy of Sciences, Beijing 100101, China; State Key Laboratory of Epigenetic Regulation and Intervention, Institute of Biophysics, Chinese Academy of Sciences, Beijing 100101, China; College of Life Sciences, University of Chinese Academy of Sciences, Beijing 100049, China; State Key Laboratory of Epigenetic Regulation and Intervention, Institute of Biophysics, Chinese Academy of Sciences, Beijing 100101, China; College of Life Sciences, University of Chinese Academy of Sciences, Beijing 100049, China; State Key Laboratory of Epigenetic Regulation and Intervention, Institute of Biophysics, Chinese Academy of Sciences, Beijing 100101, China; National Laboratory of Biomacromolecules, CAS Center for Excellence in Biomacromolecules, Institute of Biophysics, Chinese Academy of Sciences, Beijing 100101, China; Shandong First Medical University & Shandong Academy of Medical Sciences, Jinan 250117, China; State Key Laboratory of Epigenetic Regulation and Intervention, Institute of Biophysics, Chinese Academy of Sciences, Beijing 100101, China; College of Life Sciences, University of Chinese Academy of Sciences, Beijing 100049, China

**Keywords:** Mitochondrial DNA, mtDNA variant, NUMT, mtDNA–nDNA variant association, Whole-genome sequencing

## Abstract

Dysfunctional mitochondria are implicated in various diseases, but comprehensive characterization of mitochondrial DNA (mtDNA) in the Chinese population remains limited. Here, we conducted a systematic analysis of mtDNA from 7331 samples, comprising 4129 Chinese samples from the NyuWa cohort and 3202 samples from the 1000 Genomes Project (1KGP). We identified 7216 high-quality mtDNA variants, which classified 7266 samples into 22 macro-haplogroups, and detected 1466 nuclear mitochondrial DNA segments (NUMTs). Among these, 88 mtDNA variants and 642 NUMTs were specific to NyuWa. Genome-wide association analyses revealed significant correlations between 12 mtDNA variants and 199 nuclear DNA (nDNA) variants. Our findings demonstrated that all individuals in both NyuWa and 1KGP harbored common NUMTs, while one-fifth possessed ultra-rare NUMTs that tended to insert into nuclear gene regions. Notably, rare NUMTs in the NyuWa cohort showed significant enrichment of nuclear breakpoints in long interspersed nuclear elements (LINEs) compared to 1KGP. Overall, this study provides the first comprehensive profile of NUMTs in the Chinese population and establishes the most extensive resource of Chinese mtDNA variants and NUMTs to date based on high-depth whole-genome sequencing, providing valuable reference resources for genetic research on mtDNA-related diseases.

## Graphical Abstract

**Figure:**
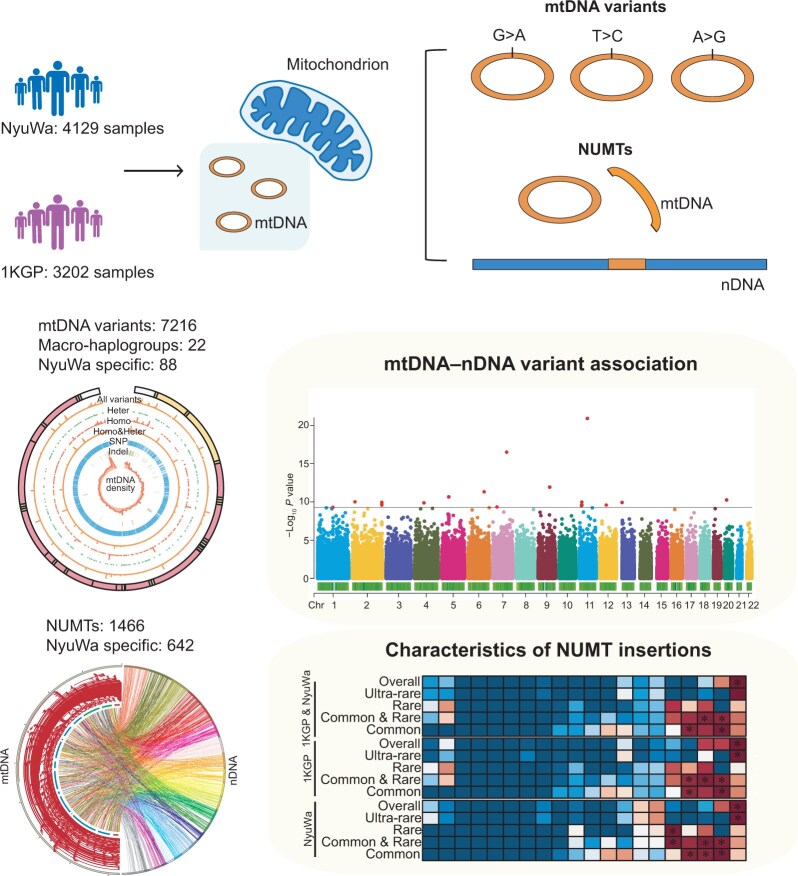

## Introduction

Mitochondria are essential organelles that generate the majority of energy for the organism through oxidative phosphorylation in mammalian cells, while their genetic material also exerts strong selective effects on the nuclear genome [1,2]. Human mitochondrial DNA (mtDNA) is a circular double-stranded DNA molecule of 16,569 bp, containing 22 transfer RNA (tRNA) genes, 2 ribosomal RNA (rRNA) genes, 13 protein-coding genes, and other non-coding regions (D-loop and intergenic regions) [1]. mtDNA is characterized by its polyploid nature, since each cell contains thousands of mitochondria, with each mitochondrion containing multiple copies of mtDNA [1].

mtDNA has a high mutation rate due to several factors: lack of DNA damage repair mechanisms, high oxidation environment, increased replication times, maternal inheritance, and lack of recombination [1,3,4]. This elevated mutation rate is associated with regional mtDNA variations that reflect prehistoric human migration patterns [5]. Heteroplasmy, the variation in the intracellular percentage of normal and mutant mtDNA, can be associated with phenotypic heterogeneity in mtDNA diseases [6,7]. The heteroplasmic level can accumulate gradually with age, and when it exceeds a critical threshold, a corresponding phenotype may occur [8,9]. mtDNA variants have been associated with a variety of complex diseases, including neurological diseases [10,11], metabolic disorders [3,12,13], and cancers [14].

Nuclear mitochondrial DNA segments (NUMTs) represent fragments of mtDNA that have integrated into the nuclear genome. Advances in whole-genome sequencing (WGS) have revealed NUMTs of varying sizes across diverse eukaryotes [1]. These NUMT insertion events occur at a rate of approximately once every 10000 human births [15]. NUMTs display non-random genomic distribution patterns and their insertion may disrupt protein-coding genes depending on locations [15,16]. Documented associations between NUMTs and human diseases include mucolipidosis IV [17], Pallister-Hall syndrome [18], Usher syndrome type IC [19], plasma factor VII deficiency [20], and cancers [15,21].

Population-specific genomics are fundamental for genetic disease research and precision medicine. Mitochondria participate in numerous biological processes, including apoptosis [22,23], lifespan modulation [23], cytoplasmic calcium buffering [24], innate immunity [25], and cell cycle [26]. While large-scale mtDNA studies like the 1000 Genomes Project (1KGP) and Genome Aggregation Database (gnomAD) have focused primarily on European populations [15,27–30], data from East Asian populations, particularly Chinese cohorts, remain limited. Human migrations have created distinct geographic distributions of mtDNA macro-haplogroups [31]. Besides, research has shown major differences in the frequency and distribution of NUMTs between different ethnic groups, with the most obvious difference observed in East Asia [15]. Our previous work with the NyuWa cohort has demonstrated its value for identifying population-specific variants [32–34].

To address this gap in global genetic diversity, we performed a comprehensive analysis of 7331 samples (with emphasis on Chinese individuals) to characterize mtDNA variants and NUMTs. This study provides the most extensive high-depth WGS resource of Chinese mtDNA variants and NUMTs to date.

## Results

### 7216 high-quality mtDNA variants and 1466 distinct NUMTs

We conducted a comprehensive characterization of mtDNA from two cohorts: the NyuWa dataset (4129 Chinese individuals) and the 1KGP dataset (3202 samples) (Table S1). The NyuWa samples were collected from 24 administrative divisions in China, including 17 provinces, 3 autonomous regions, and 4 municipalities. Most samples originated from East, North, and South China, with significant representation from Shanghai, Guangdong, and Beijing. The cohort predominantly comprised Han Chinese, as ethnic minorities are geographically clustered and underrepresented in the sampling regions. Based on sex chromosome coverage, the cohort included 1787 females and 2342 males. The 1KGP dataset represented five continental populations: Europe (EUR), East Asia (EAS), South Asia (SAS), Africa (AFR), and the Americas (AMR) (Table S1).

Using WGS data, we identified mtDNA variants through the mitochondrial variant calling pipeline [27] (see Materials and methods) and the non-reference NUMTs via the NUMT-detection method [15] across 7331 WGS samples (NyuWa and 1KGP). The median nuclear DNA (nDNA) depth was 31× (NyuWa) and 33× (1KGP) (Figure S1A), while the median mtDNA depth was ∼ 3000× (NyuWa) and ∼ 12,000× (1KGP) (Figure S1B). To filter out potential contaminated samples, three methods were used: assessment of mtDNA copy number, assessment of nDNA contamination by VerifyBamID2, and assessment of mtDNA contamination by Haplocheck [35] (Figure S1C). After filtering out duplicate data, 7266 samples were retained for mtDNA variant analysis, revealing a weak correlation between nuclear and mtDNA depth (Figure S1D). For NUMT profiling, we removed samples based on insert size, retaining 7324 samples.

**Figure 1:**
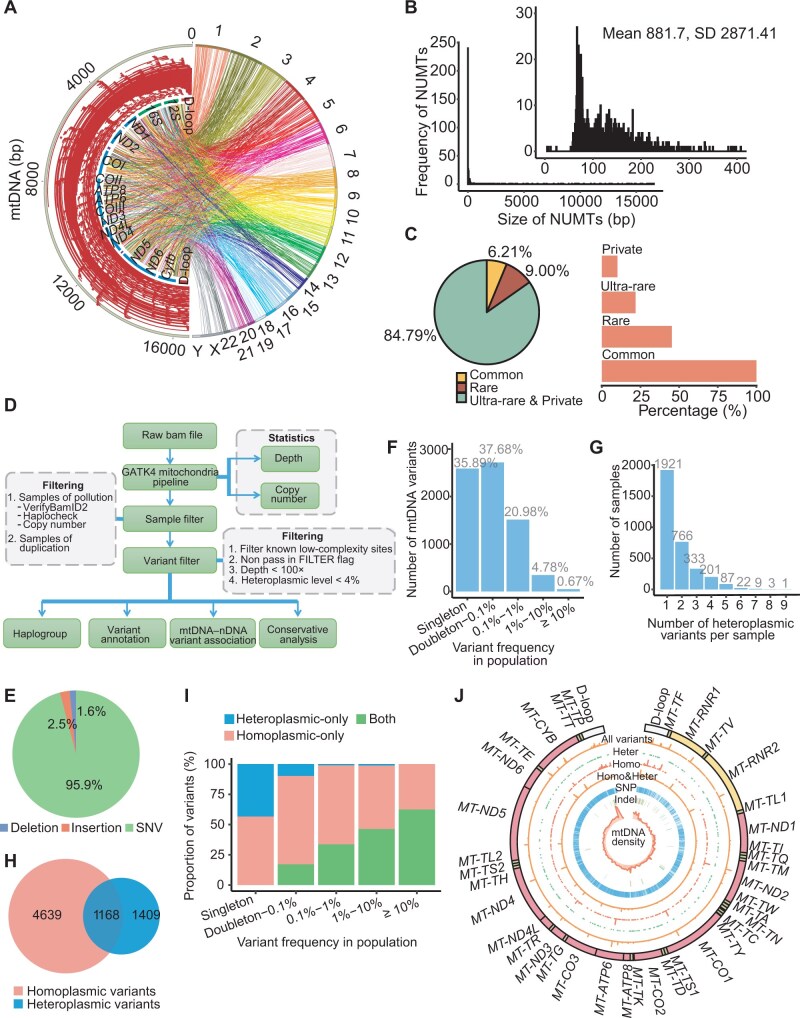
Characteristics of mtDNA variants and NUMTs identified in this study **A**. Circos plot of all NUMTs. Left, size and location of NUMTs on mtDNA. Right, chromosomal locations of NUMT insertions. **B**. Size distribution of NUMTs. NUMTs less than 400 bp in length are shown in the inset. **C**. Left, proportion of NUMTs stratified by population frequency. Right, proportion of samples carrying common, rare, ultra-rare, and private NUMTs. **D**. Pipeline of mtDNA variant calling and filtering. **E**. Variant type (SNV, deletion, and insertion) composition of distinct high-quality mtDNA variants in this study. **F**. Number of high-quality mtDNA variants detected at different variant frequencies in this study. **G**. Number of heteroplasmic variants per sample in this study. **H**. Number of homoplasmic and heteroplasmic high-quality mtDNA variants and their overlap in this study. **I**. Proportion of variants (homoplasmic-only, heteroplasmic-only, and both) at different variant frequencies. “Both” denotes variants observed at both the homoplasmic and heteroplasmic levels. **J**. The mtDNA variant spectrum of this study. From outer circle to inner circle: mitochondrial genome, mtDNA variants, heteroplasmic variants, homoplasmic variants, homoplasmic and heteroplasmic variants, SNPs, indels, and mtDNA variant density. mtDNA, mitochondrial DNA; NUMT, nuclear mitochondrial DNA segment; SD, standard deviation; SNV, single-nucleotide variant; indel, insertion and deletion; SNP, single-nucleotide polymorphism.

Using stringent filtering (≥5 discordant read pairs), we detected 120,400 non-reference NUMTs (**Figure 1**A), with a mean of 16.44 NUMTs per sample [standard deviation (SD) = 11.91]. Grouping NUMT clusters from multiple samples yielded 1466 distinct NUMTs, ranging from 5 bp to 16,568 bp (median: 119 bp; mean: 881.7 bp; SD = 2871.41) (Figure 1B). Compared with the 100,000 Genomes Project (63.2% of NUMTs < 200 bp; 77.8% < 500 bp) [15], our dataset had a higher proportion of short insertions (88.51% of NUMTs < 400 bp; 89.15% < 500 bp) (Figure 1B). All NUMTs were divided into four categories based on population frequency: common [frequency (F) ≥ 1%], rare (0.1% ≤ F < 1%), ultra-rare (F < 0.1%), and private (detected in only one person). Common and rare NUMTs accounted for 6.21% and 9.00% of the dataset, respectively, while the majority of NUMTs (1243, 84.79%) were ultra-rare or private (Figure 1C).

NUMTs can lead to false-positive variant calls at low variant allele frequencies (VAFs) and may also result in misinterpretation of genuinely homoplasmic variants as heteroplasmic. To reduce false positives caused by NUMTs, we filtered out the samples with low mtDNA copy number which may prone to cause NUMT misalignment [27], and the depth ratio distribution of nDNA to mtDNA was less than 0.04 (4/100) in all samples (Figure 1D, Figure S1E). That is, if the proportion of reads supporting a variant at the locus exceeded 0.04 (4/100), the locus was more likely to be a genuine mtDNA variant than a NUMT insertion. Based on this, to obtain a high-confidence variant set, we filtered out variants with VAF < 0.1, and the results showed that each sample contained at least 2 mtDNA variants, with a maximum of 101 and a median of 36 (Figure S1F).

The NyuWa and 1KGP datasets collectively contained 268,746 mtDNA variants in total at varying heteroplasmic levels: 97.8% were homoplasmic (VAF ≥ 0.95), and 2.2% were heteroplasmic (VAF: 0.1–0.95) (Figure S2A), consistent with gnomAD (98% homoplasmic and 2% heteroplasmic). After deduplication, we finally obtained 7216 high-confidence mtDNA variants from 7266 individuals. Single-nucleotide variants (SNVs) dominated (95.9%) (Figure 1E), which were mainly transitions (Figure S2B), especially G>A and T>C (Figure S2C), consistent with the reported mtDNA variation signature in both population-based studies [27,28] and cancer studies [36,37]. As for insertions and deletions (indels), most were 1-bp indels (Figure S2D). Most of the mtDNA variants were detected at low population frequencies in NyuWa and 1KPG (Figure 1F), with 47.97% (142/296) of indels being singletons (Figure S2E). Heteroplasmic variants were detected in 3343 (46.01%) samples (Figure 1G), with ∼ 80.38% harboring one or two heteroplasmic variants. Variant distribution analysis revealed 1409 (19.53%) heteroplasmic-only and 4639 (64.29%) homoplasmic-only variants in NyuWa and 1KGP, which were higher proportions compared to gnomAD (15% heteroplasmic-only and 48% homoplasmic-only) (Figure 1H), suggesting potential population-specific differences or differences in sequencing sensitivity between studies. Most insertions fell into the heteroplasmic-only category (Figure S2F). In addition, heteroplasmic variants tended to be at low population frequencies, while the homoplasmic variants spanned a relatively wider population frequency spectrum (Figure 1I). Notably, 79.77% of heteroplasmic-only variants were singletons (Figure S2G), suggesting that these variants may be newly generated or harmful to the organism. The map of all mtDNA variants in NyuWa and 1KGP is shown in Figure 1J, including the distribution of variant population frequency and the density of variants.

In order to sort out the mtDNA variants and NUMTs obtained in this study, we developed the NyuWa mtDNA Variant Resource (NMVR) website (http://bigdata.ibp.ac.cn/NMVR/), enabling users to query mtDNA variants and NUMTs of interest. NMVR integrates variant data from NyuWa, gnomAD, 1KGP, and HelixMTdb for comprehensive comparisons.

### Specific mtDNA variants and NUMTs in NyuWa

**Figure 2:**
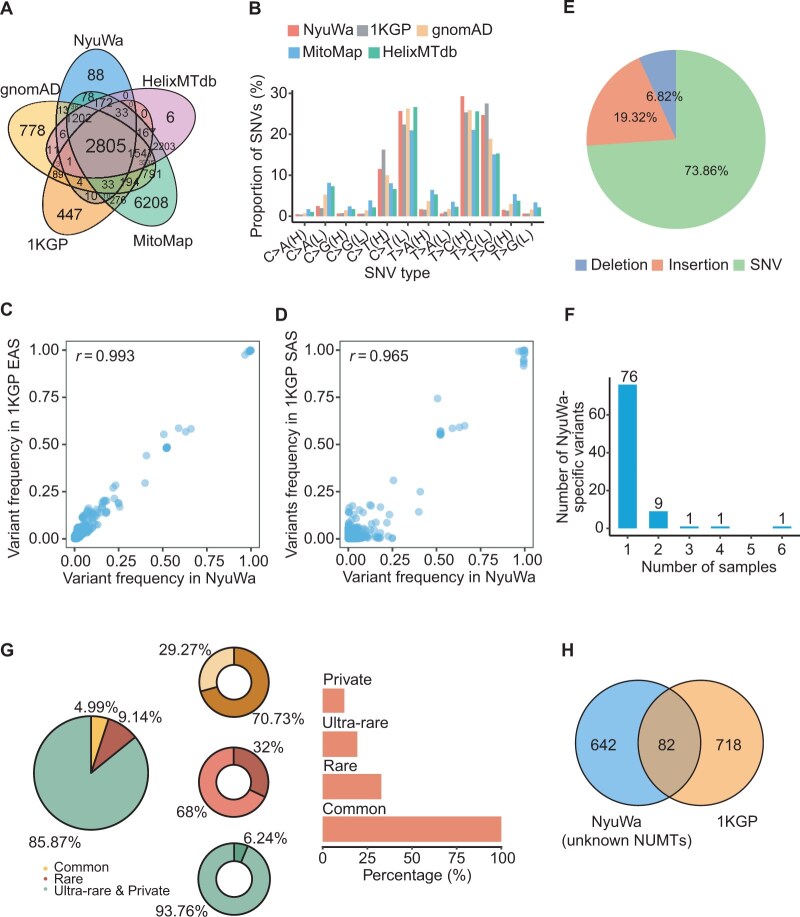
Comparison of mtDNA variants and NUMTs in NyuWa with other resources **A**. Overlap of variants (referring to all mtDNA variants, including homoplasmic variants, heteroplasmic variants, and variants detected at both homoplasmic and heteroplasmic levels) of NyuWa and other mtDNA variant resources. **B**. SNV types of different cohorts. H represents the heavy strand and L represents the light strand of the double-stranded mitochondrial DNA. **C**. Variant frequencies of shared mtDNA variants in NyuWa and 1KGP EAS in this study. Pearson correlation coefficient (*r*) is shown. **D**. Variant frequencies of shared mtDNA variants in NyuWa and 1KGP SAS in this study. Pearson correlation coefficient (*r*) is shown. **E**. Variant type composition of NyuWa-specific mtDNA variants. **F**. Number of samples for NyuWa-specific variants. **G**. Left, proportion of NUMTs stratified by population frequency. Middle, proportion of reported (darker color) and newly identified (lighter color) NUMTs. Right, proportion of samples carrying NUMTs stratified by population frequency. **H**. Venn diagram comparing unknown NUMTs in NyuWa and NUMTs in 1KGP. EAS, East Asia; SAS, South Asia.

In order to explore shared and specific mtDNA variants and NUMTs, we compared NyuWa mtDNA resource with other resources. Of 4493 NyuWa mtDNA variants (including homoplasmic-only, heteroplasmic-only, and variants detected at both homoplasmic and heteroplasmic levels), 4405 (98%) were shared with at least one other database (**Figure 2**A), indicating the reliability of all mtDNA variants in our call set. The SNV types of different cohorts were also similar (Figure 2B). For shared variants, NyuWa exhibited strong population frequency correlations with other resources (Figure S3A–C). Notably, EAS and SAS subpopulations from the 1KGP showed higher concordance with NyuWa than other groups (Figure 2C and D, Figure S3D–F). SNVs exhibited relatively higher consistency when stratified by variant types (Figure S3G–I). In addition, there were 88 novel variants, named NyuWa-specific mtDNA variants, of which 73.86% were SNVs (Figure 2E). Over 50% of these variants localized to protein-coding genes and were detected at both homoplasmic and heteroplasmic levels (Figure S3J and K). As expected, these NyuWa-specific variants exhibited low population frequencies, with 76 of the 88 variants (86.4%) being singletons (Figure 2F). The position of m.8273CCCCTCT>C, a NyuWa-specific variant observed in 6 individuals, overlapped with m.8273_8281del which was reported to be associated with maternally inherited essential hypertension (MIEH) in China [38].

Among the 821 distinct NUMTs identified in the NyuWa cohort, 705 NUMTs (85.87%) were ultra-rare and private variants, carried by 19.36% and 12.11% of the individuals, respectively (Figure 2G). Common NUMTs accounted for the smallest proportion (4.99%), with most having been reported previously (Figure 2G; see Materials and methods) [15,39–42]. In contrast, only a minority of rare NUMTs were known (Figure 2G). Notably, we identified 642 Chinese-specific NUMTs in NyuWa by comparing the unknown NUMTs in NyuWa to the NUMTs in 1KGP (Figure 2H).

### Functional annotation and pathogenicity of mtDNA variants in NyuWa and 1KGP

**Figure 3:**
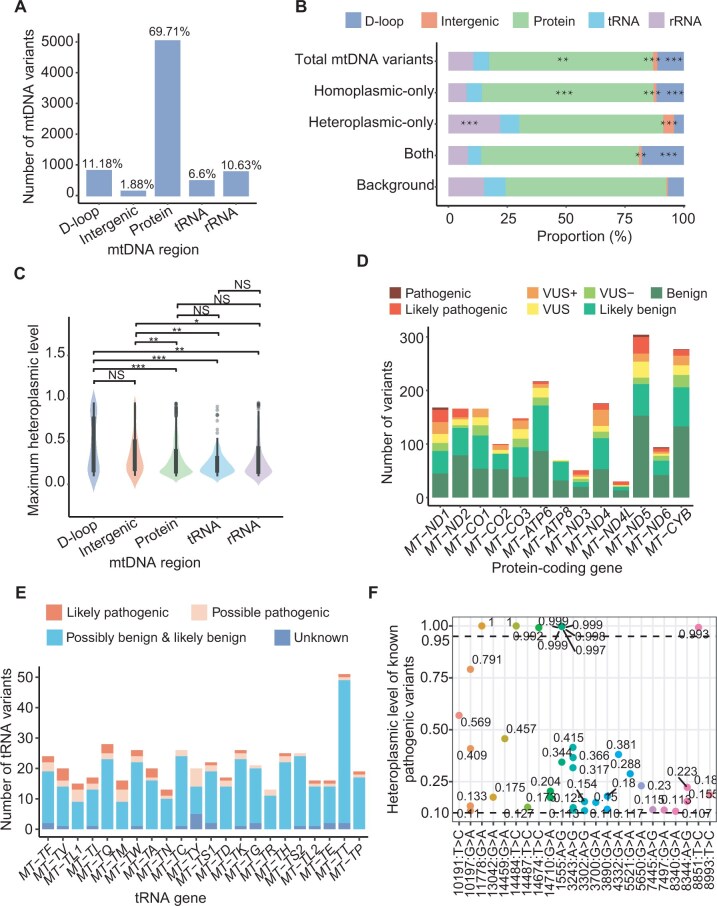
Predicted functions and pathogenicity of mtDNA variants **A**. Number of mtDNA variants annotated to distinct mtDNA regions. **B**. Proportion of variant annotations in mtDNA regions. “Both” stands for variants identified at both the homoplasmic and heteroplasmic levels. “Background” stands for the reference mtDNA genome. **, *P* ≤ 0.01; ***, *P* ≤ 0.001 (one-tailed Hypergeometric test). **C**. Distribution of the maximum heteroplasmic levels of mtDNA variants in mtDNA regions. **, *P* ≤ 0.01; ***, *P* ≤ 0.001; NS, no significance (one-tailed Wilcoxon test). **D**. Number of missense variants in protein-coding genes. APOGEE 2 refers to five pathogenicity classes: benign, likely benign, VUS, likely pathogenic, and pathogenic. VUS+ means closer to the likely pathogenic threshold, and VUS− means closer to the likely benign threshold. **E**. Number of variants in tRNA genes. The severity of variants was defined by MitoTIP. Likely pathogenic denotes variants with high pathogenicity scores, indicating a strong likelihood of causing disease. Possible pathogenic denotes variants with moderate pathogenicity scores, suggesting a potential but less certain disease-causing effect. Possibly benign & likely benign denotes variants with low pathogenicity scores, unlikely to cause disease. Unknown denotes variants that do not have a MitoTIP percentile score. **F**. Known pathogenic mtDNA variants observed in this study along with the heteroplasmic levels. Each dot represents a single individual, and different colors represent different variants.

To assess the functional impacts of all mtDNA variants in NyuWa and 1KGP, variants were annotated using Variant Effect Predictors (VEP) [43] and Mitochondrial mutation Impact (MitImpact) [44]. Among high-quality mtDNA variants, 69.71% were located in protein-coding genes (**Figure 3**A). Most indels (186/296) occurred in D-loop and intergenic regions, which typically have weaker functional impacts than those in gene regions (Figure S4A). While most heteroplasmic variants were annotated to D-loop, rRNA, and protein-coding regions (Figure S4B), those detected exclusively at heteroplasmic levels showed enrichment in rRNA and intergenic regions (Figure 3B). The maximum heteroplasmic levels in gene regions were lower than those in D-loop and intergenic regions (Figure 3C), likely because variants with high heteroplasmic levels may lead to severe functional impairment. Then, we explored the functional annotation and pathogenicity of mtDNA variants within NyuWa and 1KGP, respectively. Similar trends were observed in NyuWa and 1KGP, with most mtDNA variants located in protein-coding genes (70.38% in NyuWa and 68.96% in 1KGP, Figure S5A) and lower maximum heteroplasmic levels in gene regions compared to D-loop and intergenic regions (Figure S5B). Notably, NyuWa showed greater enrichment of heteroplasmic-only variants in D-loop and intergenic regions compared to 1KGP (Figure S5C).

Among the 5030 mtDNA variants located in protein-coding genes, the homoplasmic-only variants were widespread in all 13 protein-coding genes (Figure S4C). Moreover, 1962 of the 5030 variants were missense mutations. Pathogenicity annotation was performed using the APOGEE2 meta-predictor from the MitImpact database. Only 13 of the missense mutations were annotated as pathogenic (Figure 3D; Table S2), with 12 identified in 1KGP and one in NyuWa (Figure S5D). The NyuWa pathogenic variant, m.11778G>A in *MT-ND4*, is linked to Leber hereditary optic neuropathy (LHON), a maternally inherited mitochondrial disease causing vision loss [45]. The m.11778G>A mutation may cooperate with other mtDNA variants, raising the penetrance and expressivity of vision loss in Chinese [46].

In addition to variants in protein-coding genes, we also observed the functionally disruptive variants in tRNA genes. The pathogenicity of tRNA-associated variants was predicted based on the scoring matrix from MITOMAP [47]. Among the 476 variants annotated to tRNA genes, 31 were assigned as likely pathogenic (Figure 3E): 26 in 1KGP and 7 in NyuWa (Figure S5E). Unlike those in protein-coding genes, heteroplasmic-only variants in tRNA genes exhibited a biased distribution (Figure S4D).

In the NyuWa and 1KGP cohorts, a total of 23 known pathogenic mtDNA variants were detected in 39 individuals (Table S3), including 9 with homoplasmic variants (Figure 3F). The m.1555A>G variant, which was reported to be associated with deaf at homoplasmy, had the highest carrier frequency with 0.0015 (6/4064) compared to other known pathogenic mtDNA variants in NyuWa (Figure S4E). The prevalence of this pathogenic variant was consistent with gnomAD’s multi-population frequency (0.0013, 1/763) [27].

### 12 mtDNA variants significantly associated with nDNA variants

**Figure 4:**
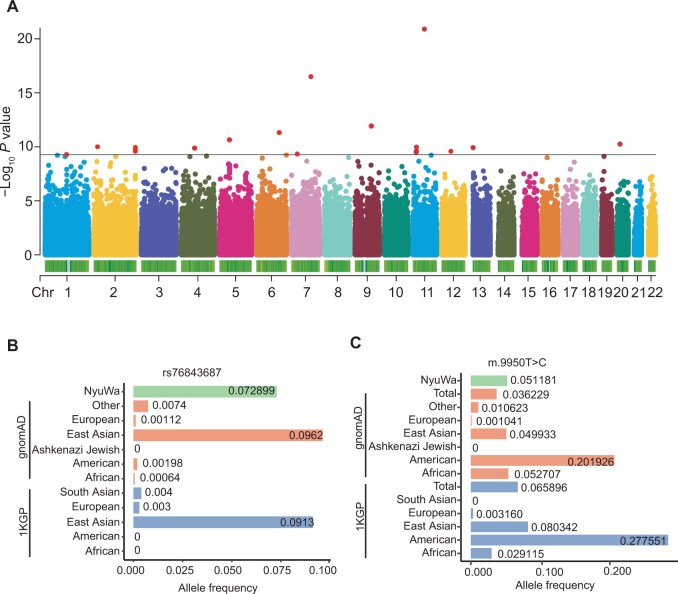
Association between mtDNA variant m.4769A>G and nDNA variants, with cross-population frequency analysis of rs76843687 and m.9950T>C **A**. Manhattan plot of the association *P* values for mtDNA variant m.4769T>C and nDNA variants. The horizontal line represents the study-wide significant threshold of 5.4 × 10^−10^. **B**. Allele frequencies of nDNA variant rs76843687 in NyuWa, gnomAD, and 1KGP. The category “Other” encompasses samples that do not belong to the listed populations (European, East Asian, Ashkenazi Jewish, American, and African in gnomAD) and includes additional populations not specifically detailed in the figure. **C**. Population/variant frequencies of mtDNA variant m.9950T>C in NyuWa, gnomAD, and 1KGP. The term “Total” refers to the 1KGP or gnomAD entire dataset.

mtDNA-encoded genes need to interact with nDNA-encoded genes to function, suggesting potential correlations between them. Previous studies have demonstrated that mtDNA–nDNA variant interactions can affect the development and progression of complex diseases, including cardiomyopathy, Parkinson’s disease, and Alzheimer’s disease [48–51]. To explore these associations, we conducted a genome-wide association analysis using 3945 unrelated samples from NyuWa, investigating 93 common mtDNA variants [minor allele frequency (MAF) ≥ 5%] and genome-wide 7,124,343 nDNA variants (MAF ≥ 5%) (see Materials and methods). After multiple comparison correction (FDR_BH < 0.05/93 = 5.4 × 10^−4^), we discovered significant associations between 12 mtDNA variants and 199 nDNA variants (Table S4). Nine of these mtDNA variants were located in protein-coding genes, all representing haplogroup markers in the Phylotree database (v17) [52]. Of the 12 mtDNA variants significantly associated with nDNA variants, five were homoplasmic (Figure S6A). Among these, m.4769A>G located in the *MT-ND2* gene showed the strongest association with nDNA variants (**Figure 4**A). This variant has been previously associated with Tic disorders (TDs) [53] and was found to interact with seven nDNA variants (rs2255074, rs1107479, rs7973157, rs2950390, rs2958154, rs2290893, and rs2035081) [54]. Notably, the nDNA variant rs76843687, which was significantly associated with m.9950T>C, occurred more frequently in East Asians than in Europeans (Figure 4B). This variant was located in the intron of the Follistatin-like protein 1 (*FSTL1*) gene. The mtDNA variant m.9950T>C, annotated to the protein-coding gene *MT-CO3*, showed relatively higher frequency in East Asians (Figure 4C). Three additional mtDNA variants (m.13759G>A, m.9180A>G, and m.9053G>A) also showed the notable frequencies in NyuWa and East Asians (Figure S6B–E). Besides, 199 nDNA variants were located within or upstream/downstream of 145 distinct genes. Upon intersecting these 145 genes with 2282 candidate nuclear genes that may contribute to mitochondrial function (MT-nDNA) [55], we identified a subset of 10 MT-nDNA genes: *ATG4D*, *MIMT1*, *NAV2*, *NUCB2*, *PECAM1*, *CALR*, *ECHDC3*, *FKBP10*, *STARD7*, and *TAT*. These 10 MT-nDNA genes function in cellular energy metabolism, autophagy, and mitochondrial homeostasis. Genes located within 25 kb upstream and downstream of the 199 nDNA variants were significantly enriched in pathways related to lipid metabolism and inflammatory response (Figure S6F). Applying a more stringent threshold (*P* = 5.0 × 10^−8^/93 = 5.4 × 10^−10^), we identified 76 significant associations, implicating 8 mtDNA variants and 59 nDNA variants.

### Haplogroup composition of the Chinese population

**Figure 5:**
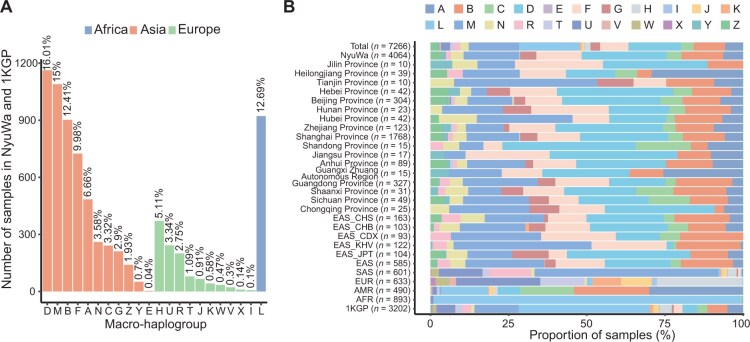
Haplogroup composition in this study **A**. Number of samples under each macro-haplogroup in NyuWa and 1KGP. Colors indicate macro-haplogroups associated with Africa, Asia, and Europe, according to the population division of MITOMAP. **B**. Composition of macro-haplogroups in each administrative region. EUR, Europe; AMR, the Americas; AFR, Africa; EAS_CHS, Han Chinese South; EAS_CHB, Han Chinese in Beijing; EAS_CDX, Chinese Dai in Xishuangbanna; EAS_KHV, Kinh in Ho Chi Minh City, Vietnam; EAS_JPT, Japanese in Tokyo.

The maternal inheritance and non-recombining nature of mtDNA enable haplogroup classification based on characteristic variant sets. Haplogroup analysis is essential for mtDNA variant studies, providing critical insights into the genetic lineage and historical population migration [56]. Also, it is crucial for correctly understanding the function of mtDNA variants, especially in disease and population genetics [57]. By constructing haplogroups, we can trace the inheritance patterns of mtDNA variants, identify Chinese population-specific frequency spectra, and reveal heterogeneity across different geographic regions in China. Based on the Phylotree database (v17) [52], the most likely haplogroup of each individual was assigned using Haplogrep [58]. Among the 5184 haplogroups included in the Phylotree database [52], a total of 1563 haplogroups were found in NyuWa and 1KGP (Figure S7A). Quality scores greater than 0.8 were obtained for 99.59% of samples based on Haplogrep results (Figure S7B). The 7266 samples were classified into 22 macro-haplogroups, with 72.53% (5270/7266) belonging to Asia haplogroups based on the population divisions of MITOMAP (https://www.mitomap.org/foswiki/pub/MITOMAP/WebHome/simple-tree-mitomap-2019.pdf) (**Figure 5**A). Among the 22 macro-haplogroups, group D contained the largest proportion of samples, followed by M, B, and F of Asia haplogroups. As a descendant of macro-haplogroup M, macro-haplogroup D is believed to have arisen in Central Asia (https://haplogroup.org/mtdna/rsrs/l123456/l23456/l2346/l346/l34/l3/m/m80d/d/). Moreover, 113 samples from the NyuWa cohort belonged to European haplogroups, including 78 in macro-haplogroup R, which is a descendant of macro-haplogroup N. The macro-haplogroup R originated in West Asia [59], and now dominates the European maternal landscape, making up 75%–95% of the lineages there (https://haplogroup.org/mtdna/rsrs/l123456/l23456/l2346/l346/l34/l3/n/r/). The macro-haplogroup H, prevalent in Europe, accounted for 5.11% of the total 7266 samples (Figure 5A).

The definition of Phylotree about haplogroup determines the difference in the number of mtDNA variants among different ethnic groups [27]. We analyzed variant counts in each sample by macro-haplogroups, and found 20–50 variants per sample across all macro-haplogroups except H (Figure S7C), with similar distributions observed for homoplasmic variants (Figure S7D). However, heteroplasmic variants were relatively few in number and showed minimal variation across macro-haplogroups (Figure S7E).

For geographic analysis, we calculated macro-haplogroup proportions in Chinese administrative regions with > 10 samples (Figure S7F). Macro-haplogroups A, B, D, F, and M each exceeded a frequency of 3% in corresponding regions (Figure 5B, Figure S7G). NyuWa’s macro-haplogroup composition resembled that of 1KGP EAS (Figure 5B). In Heilongjiang Province and Guangxi Zhuang Autonomous Region, macro-haplogroup A was more prevalent, a pattern similar to that in 1KGP AMR; however, this composition may be due to the limited sample sizes. Given that the NyuWa samples were primarily sourced from Beijing, Shanghai, and Guangdong Province (with > 300 samples each), the macro-haplogroup compositions of these regions exhibited similarity. We then analyzed the mtDNA variant landscape across these three regions (Figure S8). The mtDNA variant patterns and sample distributions in Beijing, Shanghai, and Guangdong Province were similar, consistent with the overall NyuWa cohort (Figure S8A–F), although sample size differences and region-specific variants were observed (Figure S8G).

### Relatively higher stability of rRNA- and tRNA-encoding regions than other regions in mtDNA

**Figure 6:**
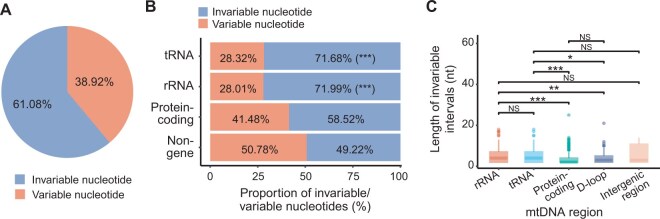
Invariable segments in mtDNA **A**. Proportion of invariable and variable nucleotides in mtDNA in the NyuWa cohort. **B**. Proportion of invariable/variable nucleotides in different mtDNA regions in the NyuWa cohort. ***, *P* ≤ 0.001 (one-tailed Hypergeometric test). **C**. Length distribution of invariable intervals in different mtDNA regions in the NyuWa cohort. *, *P* ≤ 0.05; **, *P* ≤ 0.01; ***, *P* ≤ 0.001; NS, no significance (one-tailed Wilcoxon test).

To identify specific or highly conserved regions that are invariant across the Chinese population, we extracted regions with no mtDNA variants in the NyuWa cohort as previously described [28]. Considering only SNVs and deletions which alter existing nucleotides in the mtDNA sequence, 61.08% of mtDNA nucleotides were invariable and thus relatively stable in this cohort (**Figure 6**A). These stable nucleotides showed higher conservation than variable sites (Figure S9A and B). Furthermore, rRNA- and tRNA-encoding regions had a higher proportion of invariable nucleotides than protein-coding and non-gene regions (Figure 6B). When considering consecutive invariant sequences (including insertions), we detected 2503 invariant intervals (> 1 nt), with 60 exceeding 10 nt (Figure S9C). In this cohort, rRNA and tRNA genes harbored longer invariable intervals compared with other regions (Figure 6C), though the longest invariable interval occurred in the protein-coding gene, *MT-ND5*. To explore relatively stable segments shared across different populations, we performed an integrated analysis of invariable intervals in NyuWa, gnomAD, and HelixMTdb. This analysis revealed seven shared invariable intervals (> 10 nt) localized to two rRNA genes, four tRNA genes, and one intergenic region (Table S5), confirming greater stability of rRNA- and tRNA-encoding regions compared to other genomic segments in mtDNA.

### Characteristics of NUMT insertions

**Figure 7:**
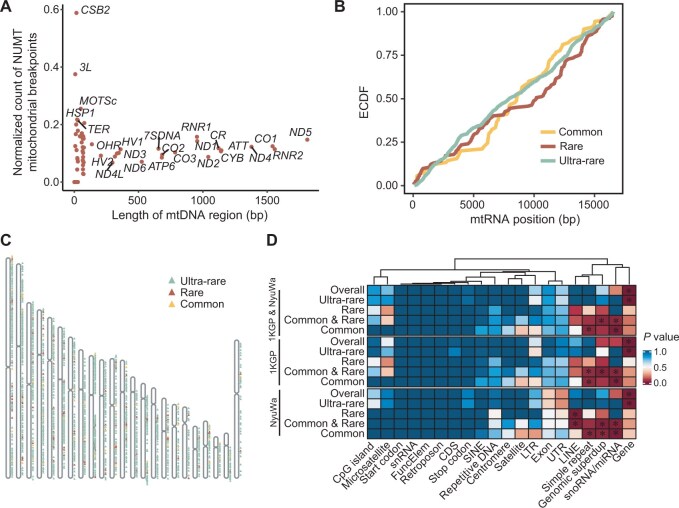
Characteristics of NUMT insertions **A**. Comparison of normalized counts of NUMT mitochondrial breakpoints across mtDNA functional regions. Normalized counts in regions with smaller intervals are greatly affected by randomness and are not considered here. **B**. ECDF plot of NUMT mitochondrial breakpoints. **C**. Chromosome map showing the chromosomal locations of NUMT insertions. **D**. *P* values for enrichment analysis of different genome regions. “Common & Rare” refers to a combined group that includes both common and rare NUMTs. *, *P* ≤ 0.05 (the permutation test). ECDF, empirical cumulative distribution function; snRNA, small nuclear RNA; FuncElem, functional element; CDS, coding sequence; SINE, short interspersed nuclear element; LTR, long terminal repeat retrotransposon; UTR, untranslated region; LINE, long interspersed nuclear element; superdup, superduplication; snoRNA, small nucleolar RNA; miRNA, microRNA.

NUMTs can be derived from any part of the mtDNA, while there is no consensus on the mechanism by which fragmented mtDNA exits mitochondria and inserts into the nuclear genome [60]. Here, we analyzed NUMT breakpoint enrichment patterns in both mitochondrial and nuclear genomes. Usually, one NUMT corresponds to two mitochondrial breakpoints. The filtered mitochondrial breakpoints were likely to involve *ND5* and *RNR1* regions (**Figure 7**A). *RNR1*, encoding rRNA, exhibited higher normalized NUMT mitochondrial breakpoint counts than other similarly-sized regions. *ND5*, encoding NADH-ubiquinone oxidoreductase chain 5, is a known mutational hotspot [61], with its extensive length contributing to a greater number of NUMT mitochondrial breakpoints. Breakpoints of common NUMTs were enriched in the middle of the mtDNA compared to those of rare NUMTs (Figure 7B). Similarly, different categories of NUMTs have different characteristics at their insertion positions in the nuclear genome, which are distributed across all chromosomes (Figure 7C). Overall, all datasets (NyuWa, 1KGP, and 1KGP & NyuWa) showed consistent enrichment patterns: common NUMT breakpoints favored genomic duplications, simple repeats, microRNA (miRNA) regions, and small nucleolar RNA (snoRNA) regions, while rare NUMT breakpoints accumulated in LINEs (Figure 7D). As ultra-rare NUMTs predominated, overall breakpoint enrichment was consistent with ultra-rare NUMT breakpoints, mainly concentrated in gene regions (Figure 7D). Notably, the NyuWa dataset revealed significant rare NUMT breakpoint enrichment in LINEs (*P* = 0.004) (Figure 7D). We also selected Beijing, Shanghai, and Guangdong Province as representative regions to illustrate the landscape of NUMTs across different administrative regions (Figure S10). While the NUMT landscapes in these regions shared similarity, which were consistent with the overall trends observed in the entire NyuWa dataset, each region also harbored specific NUMTs.

## Discussion

WGS data can be used not only for the analysis of nuclear genome variation, but also for the discovery of mtDNA variation and NUMTs [1,15]. Growing evidence links mtDNA variants to various diseases [3,10–14]. NUMTs, though typically regarded as neutral polymorphisms [62], have also been associated with human diseases [17–20], multiple cancers [14,15,21], and lifespan [16], and they may introduce false positives in mtDNA variant datasets [48]. Here, we comprehensively analyzed mtDNA variants using deep WGS data from 7266 individuals (4064 samples from NyuWa [32] and 3202 samples from 1KGP), and characterized the landscape of NUMTs across 7324 samples (4122 from NyuWa [32] and 3202 from 1KGP). We detected 7216 distinct high-quality mtDNA variants with VAF ≥ 0.1, predominantly homoplasmic variants, consistent with previous findings [27]. We found 88 NyuWa-specific mtDNA variants by comparing with other mtDNA variant resources. Given reported mtDNA–nDNA interactions in human diseases [48], we performed genome-wide association analysis in the Chinese population, identifying 12 mtDNA variants significantly associated with 199 nDNA variants. While these findings provide valuable references for mtDNA–nDNA associations, further investigation is needed to elucidate their biological significance. However, in the previous association analysis of mtDNA and nDNA in a Japanese population, no significant association was found between mtDNA and nDNA [29]. This may be due to the limited number of samples. There are 1928 individuals in the Japanese cohort while 4064 in NyuWa. On the other hand, it may also be related to the stratification of the population.

The variant m.4769A>G exhibited the most significant association with nDNA variants. Jiang et al. previously demonstrated its significant association with TDs based on an additive model [53]. Additionally, three additional mtDNA variants identified in our NyuWa mtDNA–nDNA association analysis (m.14766C>T, m.7028C>T, and m.750A>G) were also reported by Jiang et al. to associate with TDs. Willsey et al. reported nDNA variants significantly associated with TDs [63]. The nuclear genes hosting these nDNA variants overlapped with those harboring the nuclear single-nucleotide polymorphisms (SNPs) significantly associated with m.4769A>G, m.14766C>T, m.7028C>T, and m.750A>G, such as *DYNC2H1*, *HHIPL2*, *KIAA1549L*, *NAV2*, and *SLC1A3*. After annotation, 199 nDNA variants were located within or in close proximity to 145 distinct genes. From the intersection of the 145 candidate genes and the known 2282 MT-nDNA genes [55], a key subset of 10 genes emerged. These genes play established roles in cellular energy metabolism, autophagy, and mitochondrial homeostasis. Separately, genomic regions flanking the 199 nDNA variants (within 25 kb) were found to be significantly enriched for genes participating in lipid metabolism and inflammatory response pathways. This suggests that these genes might play an important role in regulating lipid levels, cholesterol storage, and the biosynthesis of inflammatory mediators. It has been previously suggested that mtDNA mutations are closely tied to lipid homeostasis [64], cholesterol metabolism [65], and inflammation [66]. These mutations can potentially lead to metabolic disorders. Further studies of the mechanisms and functions of mtDNA–nDNA variant interactions will advance our understanding of mitochondrial disease pathogenesis and potentially inform new diagnostic and therapeutic approaches.

For haplogroup analysis, we identified 1563 distinct haplogroups among the 5184 haplogroups included in the Phylotree database and classified 7266 samples into 22 macro-haplogroups, with 72.53% (5270/7266) assigned to Asian haplogroups. The most prevalent macro-haplogroups were D, M, B, and F, reflecting the significant genetic diversity within the Asian population. Notably, macro-haplogroup D, a descendant of macro-haplogroup M believed to have originated in Central Asia, was the most common. The geographic distribution of haplogroups within China revealed distinct patterns. Macro-haplogroups A, B, D, F, and M accounted for more than 3% of the samples in several administrative regions, and we also showed the landscape of mtDNA variants in Beijing, Shanghai, and Guangdong Province. However, the haplogroup composition in some regions may have been influenced by limited sample sizes. Our study highlights the rich genetic diversity of mtDNA haplogroups in the Chinese population and provides a view of their geographic distribution and ancestral origins.

It has been found that heteroplasmies throughout the mtDNA are common in normal human cells, and moreover, the frequencies of the heteroplasmic variants vary considerably between different tissues in the same individual [67]. In NyuWa, mtDNA was exclusively extracted from blood samples [32], without inclusion of mitochondria-rich tissue samples. The mtDNA variant spectrum of this Chinese cohort can only reflect the heterogeneity level in blood.

Our analysis identified 120,400 NUMTs across 1KGP and NyuWa. After deduplication among 7324 samples, we detected 1466 distinct NUMTs. These NUMTs varied in size from 5 bp to the entire mtDNA, excluding concatenated NUMTs and other complex arrangements. The NyuWa Chinese population displayed substantial NUMT diversity, with ultra-rare NUMTs accounting for 85.87% of the total, but only appearing in 19.36% of samples. Both NyuWa and 1KGP exhibited similar nuclear breakpoint enrichment patterns. Specifically, ultra-rare NUMTs showed relatively random mitochondrial breakpoint distribution but preferentially inserted into the gene regions of the nuclear genome, potentially explaining the low population frequency. Meanwhile, mitochondrial breakpoints of common and rare NUMTs exhibited clear enrichment tendency: common NUMT breakpoints had a relatively high cumulative distribution slope in the middle mtDNA region, while rare NUMT breakpoints had a much lower slope in the same middle region. Considering the high population frequency, common NUMT nuclear breakpoints accumulated in repetitive regions, while rare NUMT nuclear breakpoints favored LINE regions. Notably, the NyuWa dataset showed significant LINE enrichment specifically for rare NUMT nuclear breakpoints (*P* = 0.004). These results differ somewhat from the 100,000 Genomes Project (predominantly European) findings [15], such as the enrichment of ultra-rare NUMT nuclear breakpoints in regulatory elements, short interspersed elements (SINEs), simple repeats, and introns. When comparing normalized breakpoint counts across functional mtDNA regions, we accounted for interval length effects, as normalized counts in regions with small intervals are greatly affected by randomness.

However, the uneven geographical distribution of samples in the NyuWa cohort limits our comprehensive analysis of regional haplogroup compositions. Therefore, increasing sample sizes from underrepresented regions would significantly improve the cohort’s representativeness.

In general, our study provides valuable reference resources for genetic research on mtDNA-related diseases, particularly in East Asian populations. We have previously established a comprehensive WGS resource for the Chinese population, laying a solid foundation for this work. In the NyuWa project, we have meticulously described the nuclear SNPs and indels [32], mobile element insertions (MEIs) [33], and short tandem repeats (STRs) [34]. Future integration of these datasets will enable more comprehensive investigation of nuclear–mitochondrial genome interactions, complex variation associations, and their roles in disease susceptibility and progression.

## Materials and methods

### Sample collection and mtDNA variant calling

A total of 7331 samples in this study were collected from NyuWa and 1KGP. Among these, 4129 samples of the NyuWa cohort [32] were from different administrative regions of China. WGS was performed using Illumina platform and the sequencing data were aligned to the GRCh38 reference genome (containing mtDNA, NC_012920.1) to get bam files using BWA-mem (v0.7.15) [68], following the GATK Best Practices Workflows [69,70]. mtDNA variants were identified for each sample using the mitochondrial variant calling pipeline (https://github.com/gatk-workflows/gatk4-mitochondria-pipeline) with default parameters [27]. The same workflow was applied for mtDNA variant calling in the 1KGP samples.

### Acquisition of high-quality mtDNA variant set

To obtain high-quality mtDNA variants, we applied separate filtration steps for samples and variants. Sample filtration was performed as follows. (1) VerifyBamID2 (v1.0.6) [71] was used to assess contamination based on nDNA, and samples with contamination > 2% were excluded. (2) Samples reported as contaminated by haplocheck (v1.3.2; a tool to identify contamination using the mitochondrial phylogeny [35]) were removed. (3) Samples with mtDNA copy number < 50 were removed [27]. The mtDNA copy number for each sample was calculated with the following formula: mtDNA copy number = (mean mtDNA depth/mean autosomal depth) × 2 [29]. (4) The duplicate data, possibly from the same persons, were discarded following previously described methods [32]. After filtration, samples used for calling mtDNA variants included 4064 Chinese samples (NyuWa) and 3202 samples (1KGP). For mtDNA variant filtering, we excluded variants in known WGS artifacts and low-complexity sites (66–71, 301, 302, 310, 316, 3107, 12,418–12,425, 16,182–16,194). The mtDNA variants which were marked as “PASS” in the final VCF file generated by mitochondrial variant calling pipeline and had the depth greater than 100× were selected. On this basis, the filtered variants with VAF (ratio of variant-supporting reads to total reads aligned to this locus) ≥ 0.1 were assigned as high-quality variants in this cohort. A total of 268,746 high-quality variants at different heteroplasmic levels were obtained. After deduplication, the final distinct high-quality mtDNA variant set contained 7216 variants derived from 4064 NyuWa samples and 3202 1KGP samples.

### Characteristic analysis of mtDNA variants

VAF was calculated as the ratio of allele depth (AD; the number of reads supporting the variant allele) to total sequencing depth (DP; the total reads covering the variant site). Using VAF values, the high-quality mtDNA variants in individuals were categorized into two groups: homoplasmic variants (VAF ranging from 0.95 to 1.00) and heteroplasmic variants (VAF ranging from 0.10 to 0.95) [27]. Homoplasmic variants indicate that the variant is present in nearly all mtDNA copies within an individual, while heteroplasmic variants reflect the coexistence of both variant and wild-type mtDNA copies. Each distinct variant was further categorized based on its observed VAF distribution across individuals: detected only at homoplasmic level, detected only at heteroplasmic level, detected both at homoplasmic and heteroplasmic levels (defined as “both” in this study). We also analyzed population-level allele frequencies to assess variant prevalence. Based on these analyses, we described the characteristics of the mtDNA variants. Circos (v0.69.5) [72] was used to visualize mtDNA variant distributions.

### Functional annotation of mtDNA variants

The annotation of mtDNA variants was performed using VEP (v101) [43], with parameters “- -everything - -flag_pick_allele - -distance 0”. For the variants in protein-coding genes, we screened for missense mutations among the 7216 high-quality mtDNA variants and performed pathogenicity annotation based on the APOGEE2 predictor results from the MitImpact database (v3.1.2) [44]. For variants in tRNA genes, we classified pathogenicity based on MitoTIP [47] scores, which was obtained by extracting and submitting the related tRNA variants to the Mitomaster Web API (https://www.mitomap.org/foswiki/bin/view/MITOMASTER/WebHome) [28]. The tRNA variants were classified as “likely pathogenic” with score > 16.25 (75%–100%), “possibly pathogenic” with score = 12.66–16.25 (50%–75%), “possibly benign” with score = 8.44–12.66 (25%–50%), and “likely benign” with score < 8.44 (0%–25%).

### Comparison with other mtDNA variant resources

We obtained mtDNA variants from three public resources: gnomAD (https://gnomad.broadinstitute.org/downloads#v3-mitochondrial-dna; downloaded January 17, 2022), HelixMTdb (https://helix-research-public.s3.amazonaws.com/mito/HelixMTdb_20200327.tsv), and MITOMAP (https://www.mitomap.org/MITOMAP; downloaded December 28, 2023). After dividing multi-allele and removing duplicate variants, the final number of mtDNA variants was 10,850 from gnomAD, 11,491 from HelixMTdb, and 19,092 from MITOMAP. We compared the high-quality mtDNA variants of NyuWa mtDNA resource with these three databases and 1KGP in terms of shared variants, SNV types, and the correlation of variant frequency in populations.

### Analysis of mtDNA–nDNA variant association

To analyze the association between mtDNA and nDNA variants, we eliminated samples of possible relatives from 4064 samples using the method described in the NyuWa Genome resource [32]. This resulted in 3945 unrelated samples for the association analysis. We treated mtDNA variant heteroplasmic levels as the continuous phenotype. A total of 93 mtDNA variants with major allele frequency > 5% were chosen from the unrelated samples, and 7,090,977 nDNA variants with sample call rate > 0.9, variant call rate > 0.9, MAF > 5%, and Hardy-Weinberg equilibrium *P* ≥ 1.0 × 10^−6^ were extracted from the same samples. The analysis of mtDNA–nDNA variant association was conducted using generalized linear model (glm) by PLINK (v2.00a5.10) with top 12 principal components (PCs) of nDNA variants and sex as covariates. The sex of all samples was inferred based on chrX ploidy and chromosome depth, following the detailed method in the NyuWa Genome resource [32]. To account for multiple testing of mtDNA variants, FDR_BH < 0.05/93 = 5.4 × 10^−4^ was regarded as the threshold for significant association. A more stringent threshold was defined as *P* < 5 × 10^−8^/93, equating to 5.4 × 10^−10^ [29].

### Annotation of mtDNA haplogroups

Haplogrep [57] was used to assign haplogroups to each sample based on the Phylotree database (v17) [52]. Prior to analysis, we manually converted left-aligned indels in our cohort to right-alignment to ensure compatibility with Phylotree’s requirements. Haplogrep was executed with default parameters, with the additional specifications of ‘- -hetLevel 0.1 - -extend-report’. Only the first ranked haplogroup of each sample was reported and used for subsequent analysis. To obtain the composition of mtDNA haplogroups in different administrative regions of China, we extracted and statistically analyzed the macro-haplogroups (first letter of the haplogroup reported by haplogrep) for each individual, and compared them with 1KGP haplogroups, which were defined using the same method.

### Pathogenic mtDNA variants in NyuWa mtDNA resource

The pathogenic mtDNA variants were downloaded from MITOMAP [73] on July 27, 2021, and the variants labeled as “Confirmed” were selected. Then, 96 confirmed pathogenic variants were used to query existence, frequency, and heteroplasmic levels in NyuWa and 1KGP samples.

### Stability analysis of mtDNA regions

To analyze the invariable nucleotides in the NyuWa mtDNA resource, the variable nucleotides detected were excluded from the mtDNA sequence. The detailed method was that only SNVs and deletions, which could change the existing mtDNA nucleotides, were considered. We deduplicated the variable nucleotides and extracted the invariable nucleotides from the mtDNA sequence in NyuWa. The invariable and variable nucleotides were annotated to mtDNA regions using BEDTools intersect [74]. The PhastCons scores and phyloP scores, which were downloaded from UCSC [75], were used to evaluate the conservativeness of invariable nucleotides. The invariable nucleotides were expanded into intervals by an in-house script, and insertions were also considered. Intervals without variation were defined as invariable intervals in NyuWa. For comparative analysis, we identified NyuWa-specific invariable intervals by comparing our data with gnomAD and HelixMTdb datasets.

### NUMT detection

We detected NUMTs in blood-derived samples by analyzing WGS data from 7324 samples. Samples with insert sizes smaller than 250 bp were excluded from the analysis. Using samblaster, we processed WGS bam files to identify discordant reads and split reads. We treated discordant reads within 500 bp as a cluster. The cluster supported by less than five pairs of discordant reads are discarded, and the NUMTs within 1000 bp of each other from multiple samples were grouped as identical NUMT events. Then the split reads within 500-bp NUMT flanks (upstream 500 bp + downstream 500 bp) of each cluster were realigned to the GRCh38 reference genome using BLAT. The BLAT output was then used to identify mitochondrial and nuclear breakpoints, requiring at least two split reads for confirmation. Here we did not consider concatenated NUMTs and other complex situations.

BEDTools was used to detect known NUMTs, which were downloaded from UCSC and previous publications [15,39–42]. To search for overlaps with the NyuWa NUMT dataset, each NUMT in this published NUMT dataset was extended by 500 bp in both the upstream and downstream directions on the nuclear genome.

All steps mentioned above were performed using the public NUMT detection pipeline (https://github.com/WeiWei060512/NUMTs-detection.git) described in the 100,000 Genomes Project study [15].

### Enrichment analyses of NUMT mitochondrial and nuclear breakpoints

We examined the distribution of NUMT breakpoints across mtDNA by calculating cumulative distributions. This approach enabled us to assess the variation in breakpoint density at different mtDNA positions, thereby identifying regions with significant breakpoint enrichment.

To analyze the nuclear sequence characteristics at NUMT insertion sites, we obtained genomic feature annotations from two sources: the UCSC Genome Browser (https://genome.ucsc.edu/) and the GENCODE database (v43). The annotated features included centromeres, genomic duplications, simple repeats, functional elements, CpG islands, satellites, LINEs, SINEs, and genes. To determine the statistical significance of NUMT insertion site preferences, we conducted permutation tests. Resampling 10,000 sets of random nuclear positions within 100-bp NUMT flanks (upstream 100 bp + downstream 100 bp) was performed based on the total number of NUMTs. *P* values were then calculated by comparing with the observed NUMTs in the target regions.

### Database development

NMVR was developed by Bootstrap and PHP. Data resources were stored in the MySQL database. The resource is publicly accessible at http://bigdata.ibp.ac.cn/NMVR/, allowing users to browse and retrieve variant information.

### Statistical analysis

All statistical analyses in this study were performed using R (v4.1.0). Specific statistical tests applied in each analysis are detailed in the corresponding figure legends and subsections in Materials and methods. Statistical significance was assessed using one-sided tests unless specified otherwise.

## Ethical statement

This study was approved by the Medical Research Ethics Committee of Institute of Biophysics, Chinese Academy of Sciences (Approval No. IBP-2016-XT-1). All participants provided written informed consent. The informed consent is used to collect samples for genome studies conducted by Chinese Academy of Sciences. The consent requires participants to be 30–70-year-old patients or healthy people with full capacity. Participants voluntarily donate blood samples, provide clinical treatment information, and sign informed consent. All their personal information is kept confidential. Participants can choose not to participate in sample donation, or withdraw at any time.

## Supplementary Material

qzaf098_Supplementary_Data

## Data Availability

The DNA sequencing data of NyuWa samples generated in this study have been deposited in the Genome Sequence Archive for Human [76] at the National Genomics Data Center (NGDC), China National Center for Bioinformation (CNCB) (GSA-Human: HRA004185), and are publicly accessible at https://ngdc.cncb.ac.cn/gsa-human. These data are under restricted access for privacy protection and can be obtained by application in accordance with the “Request Data” guidance on the GSA-Human website. These data have also been deposited in the National Omics Data Encyclopedia of the Bio-Med Big Data Center, Shanghai Institute of Nutrition and Health, Chinese Academy of Sciences (NODE: OEP002803), and are publicly accessible at http://www.biosino.org/node. The user can register and login to this website and follow the guidance of “Request for Restricted Data” to request the data. The mtDNA variant file is available at the NMVR website (http://bigdata.ibp.ac.cn/NMVR). The VCF files of nDNA variants for NyuWa identified in this study have been deposited in the Genome Variation Map at the NGDC, CNCB (GVM: GVM000464). The user can contact the corresponding author to apply for permission to access this data. The alignment files for the 1KGP dataset are available at https://ftp-trace.ncbi.nlm.nih.gov/1000genomes/ftp/1000G_2504_high_coverage/data/ and https://ftp-trace.ncbi.nlm.nih.gov/1000genomes/ftp/1000G_2504_high_coverage/additional_698-_related/. Genotype data for SNPs and indels for the 1KGP dataset are available at http://ftp.1000genomes.ebi.ac.uk/vol1/ftp/data_collections/1000G_2504_high_coverage/working/20201028_3202_phased/.

## CRediT author statement

**Yuanxin Wang:** Conceptualization, Investigation, Methodology, Formal analysis, Visualization, Writing – original draft, Writing – review & editing. **Jiajia Wang:** Conceptualization, Investigation, Methodology, Formal analysis, Visualization, Writing – original draft, Writing – review & editing. **Yanyan Li:** Data curation, Methodology, Formal analysis. **Peng Zhang:** Data curation, Investigation, Methodology. **Zhonglong Wang:** Formal analysis, Visualization. **Shuai Liu:** Data curation. **Yiwei Niu:** Data curation. **Yirong Shi:** Data curation. **Sijia Zhang:** Data curation. **Tingrui Song:** Data curation. **Tao Xu:** Conceptualization, Funding acquisition, Project administration, Resources. **Shunmin He:** Conceptualization, Funding acquisition, Project administration, Resources, Writing – review & editing. All authors have read and approved the final manuscript.
