# Supplementary material for: Mitochondrial Genome Variants and Nuclear Mitochondrial DNA Segments in 7331 Individuals from NyuWa and 1KGP
Source: Genomics Proteomics Bioinformatics. 2025 Nov 5;23(5):qzaf098. doi: 10.1093/gpbjnl/qzaf098 (PMC12790922; doi:10.1093/gpbjnl/qzaf098)
Supplement: qzaf098_Supplementary_Data [file qzaf098_supplementary_data.zip › Table S2.docx]

**Table S2 Catalog of 13 pathogenic missense mutations**

| **Index** | **Symbol** | **APOGEE2_anno** | **Type** |
| --- | --- | --- | --- |
| MT_3715_G/C | *MT-ND1* | Pathogenic | missense_variant |
| MT_3733_G/C | *MT-ND1* | Pathogenic | missense_variant |
| MT_3890_G/A | *MT-ND1* | Pathogenic | missense_variant |
| MT_4142_G/A | *MT-ND1* | Pathogenic | missense_variant |
| MT_8993_T/C | *MT-ATP6* | Pathogenic | missense_variant |
| MT_11778_G/A | *MT-ND4* | Pathogenic | missense_variant |
| MT_12770_A/G | *MT-ND5* | Pathogenic | missense_variant |
| MT_13289_G/A | *MT-ND5* | Pathogenic | missense_variant |
| MT_13345_G/A | *MT-ND5* | Pathogenic | missense_variant |
| MT_13730_G/A | *MT-ND5* | Pathogenic | missense_variant |
| MT_14459_G/A | *MT-ND6* | Pathogenic | missense_variant |
| MT_14487_T/C | *MT-ND6* | Pathogenic | missense_variant |
| MT_15060_G/A | *MT-CYB* | Pathogenic | missense_variant |
