# Supplementary material for: Mitochondrial Genome Variants and Nuclear Mitochondrial DNA Segments in 7331 Individuals from NyuWa and 1KGP
Source: Genomics Proteomics Bioinformatics. 2025 Nov 5;23(5):qzaf098. doi: 10.1093/gpbjnl/qzaf098 (PMC12790922; doi:10.1093/gpbjnl/qzaf098)
Supplement: qzaf098_Supplementary_Data [file qzaf098_supplementary_data.zip › Table S3.docx]

**Table S3 Known pathogenic mtDNA variants present in 39 individuals**

| **Chr** | **Pos** | **Ref** | **Alt** | **Sample** | **ID** |
| --- | --- | --- | --- | --- | --- |
| chrM | 1555 | A | G | CN000807 | 1555:A>G |
| chrM | 1555 | A | G | CN001444 | 1555:A>G |
| chrM | 1555 | A | G | CN000722 | 1555:A>G |
| chrM | 1555 | A | G | CN004301 | 1555:A>G |
| chrM | 1555 | A | G | CN000669 | 1555:A>G |
| chrM | 1555 | A | G | CN003801 | 1555:A>G |
| chrM | 3243 | A | G | CN002517 | 3243:A>G |
| chrM | 3243 | A | G | CN002181 | 3243:A>G |
| chrM | 3243 | A | G | NA20412 | 3243:A>G |
| chrM | 3243 | A | G | CN004077 | 3243:A>G |
| chrM | 3243 | A | G | HG03371 | 3243:A>G |
| chrM | 3302 | A | G | NA06991 | 3302:A>G |
| chrM | 3302 | A | G | HG01868 | 3302:A>G |
| chrM | 3700 | G | A | HG02304 | 3700:G>A |
| chrM | 3890 | G | A | HG03606 | 3890:G>A |
| chrM | 3890 | G | A | HG03135 | 3890:G>A |
| chrM | 4332 | G | A | HG00181 | 4332:G>A |
| chrM | 5521 | G | A | HG03596 | 5521:G>A |
| chrM | 5650 | G | A | NA19445 | 5650:G>A |
| chrM | 7445 | A | G | NA18864 | 7445:A>G |
| chrM | 7497 | G | A | CN000078 | 7497:G>A |
| chrM | 8340 | G | A | HG03703 | 8340:G>A |
| chrM | 8344 | A | G | HG00132 | 8344:A>G |
| chrM | 8344 | A | G | CN000638 | 8344:A>G |
| chrM | 8851 | T | C | CN004327 | 8851:T>C |
| chrM | 8993 | T | C | NA19429 | 8993:T>C |
| chrM | 10191 | T | C | HG02871 | 10191:T>C |
| chrM | 10197 | G | A | HG03368 | 10197:G>A |
| chrM | 10197 | G | A | HG00525 | 10197:G>A |
| chrM | 10197 | G | A | HG03079 | 10197:G>A |
| chrM | 10197 | G | A | HG01605 | 10197:G>A |
| chrM | 11778 | G | A | CN001349 | 11778:G>A |
| chrM | 13042 | G | A | NA19394 | 13042:G>A |
| chrM | 14459 | G | A | HG04132 | 14459:G>A |
| chrM | 14484 | T | C | CN000091 | 14484:T>C |
| chrM | 14487 | T | C | HG02398 | 14487:T>C |
| chrM | 14674 | T | C | CN003927 | 14674:T>C |
| chrM | 14710 | G | A | HG03727 | 14710:G>A |
| chrM | 14710 | G | A | HG04080 | 14710:G>A |
