# Supplementary material for: Mitochondrial Genome Variants and Nuclear Mitochondrial DNA Segments in 7331 Individuals from NyuWa and 1KGP
Source: Genomics Proteomics Bioinformatics. 2025 Nov 5;23(5):qzaf098. doi: 10.1093/gpbjnl/qzaf098 (PMC12790922; doi:10.1093/gpbjnl/qzaf098)
Supplement: qzaf098_Supplementary_Data [file qzaf098_supplementary_data.zip › Table S5.docx]

**Table S5 Shared invariable intervals within NyuWa, gnomAD, and HelixMTdb**

| **gnomAD and HelixMTdb invariable intervals** | | | | **NyuWa invariable intervals** | | | |  |  |  |  |
| --- | --- | --- | --- | --- | --- | --- | --- | --- | --- | --- | --- |
| **Chr** | **Start** | **End** | **Region** | **Chr** | **Start** | **End** | **Region** | **Shared intervals** | **mtGene** | **mtCategory** | **Overlap** |
| chrM | 14727 | 14741 | 14 | chrM | 14727 | 14742 | 15 | 14 | *MT-TE* | tRNA | 14 |
| chrM | 5753 | 5767 | 14 | chrM | 5747 | 5772 | 25 | 14 | Intergenic | Intergenic | 7 |
| chrM | 5753 | 5767 | 14 | chrM | 5747 | 5772 | 25 | 14 | *MT-TC* | tRNA | 7 |
| chrM | 12252 | 12264 | 12 | chrM | 12250 | 12277 | 27 | 12 | *MT-TS2* | tRNA | 12 |
| chrM | 1081 | 1093 | 12 | chrM | 1082 | 1094 | 12 | 11 | *MT-RNR1* | rRNA | 12 |
| chrM | 3290 | 3304 | 14 | chrM | 3290 | 3301 | 11 | 11 | *MT-TL1* | tRNA | 14 |
| chrM | 690 | 701 | 11 | chrM | 690 | 703 | 13 | 11 | *MT-RNR1* | rRNA | 11 |

*Note*: mtGene, mtDNA genes; mtCategory, mtDNA category. The last column, “Overlap”, indicates shared intervals overlap with mtDNA genes.
