# Supplementary figures and images for: Mitochondrial Genome Variants and Nuclear Mitochondrial DNA Segments in 7331 Individuals from NyuWa and 1KGP
Source: Genomics Proteomics Bioinformatics. 2025 Nov 5;23(5):qzaf098. doi: 10.1093/gpbjnl/qzaf098 (PMC12790922; doi:10.1093/gpbjnl/qzaf098)

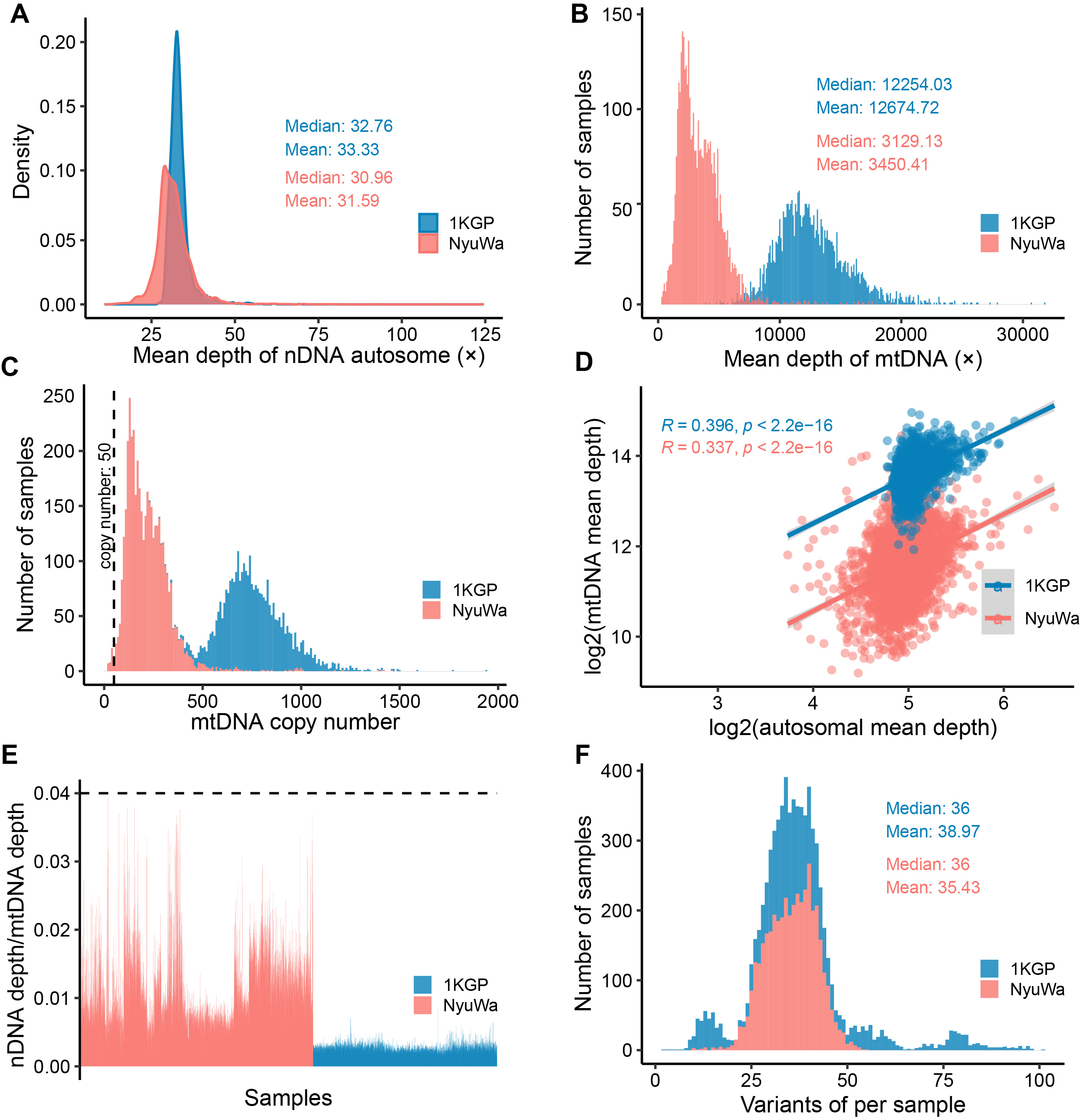

Supplement: qzaf098_Supplementary_Data [file qzaf098_supplementary_data.zip › Fig.S1.jpg]

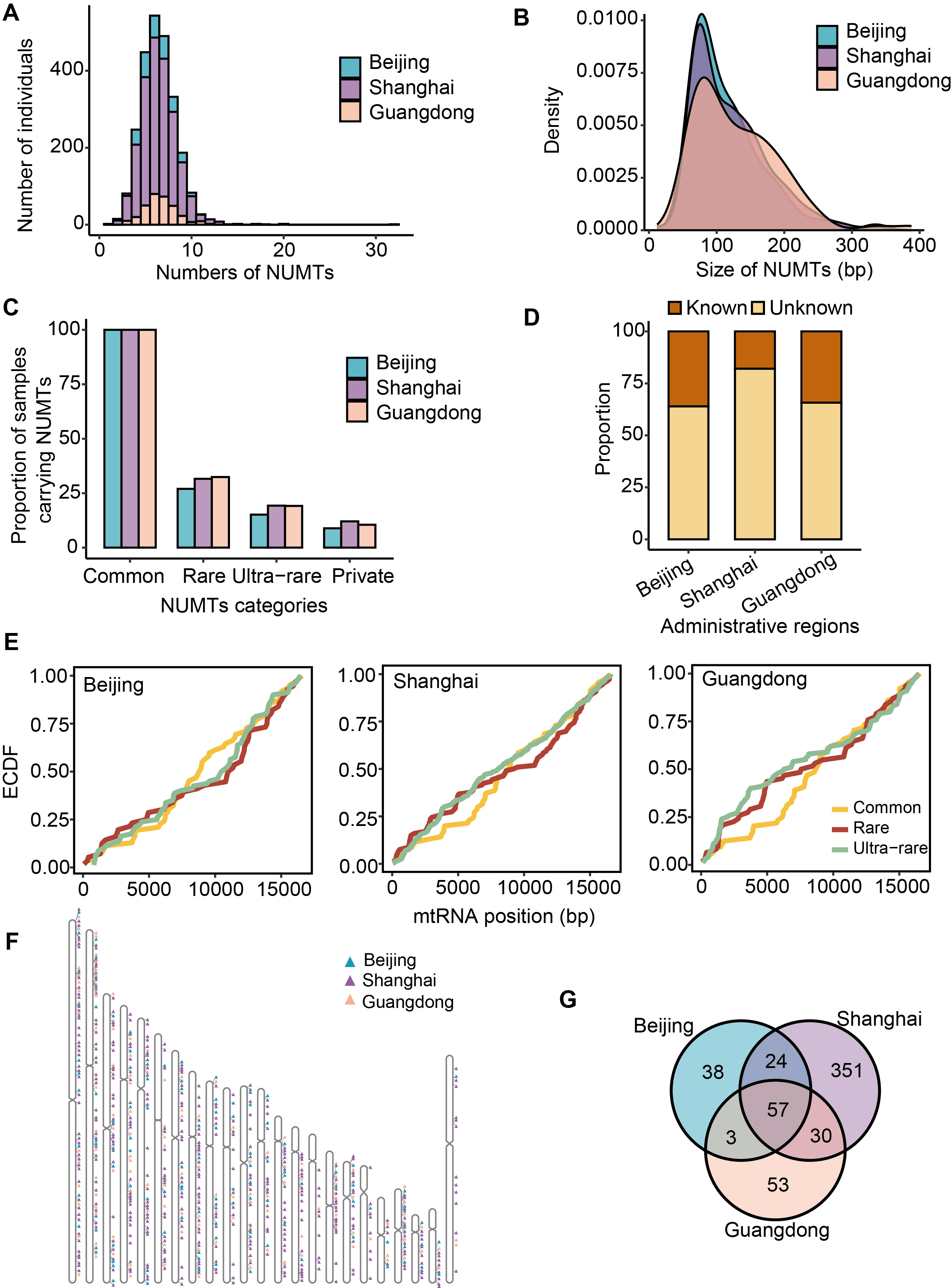

Supplement: qzaf098_Supplementary_Data [file qzaf098_supplementary_data.zip › Fig.S10.jpg]

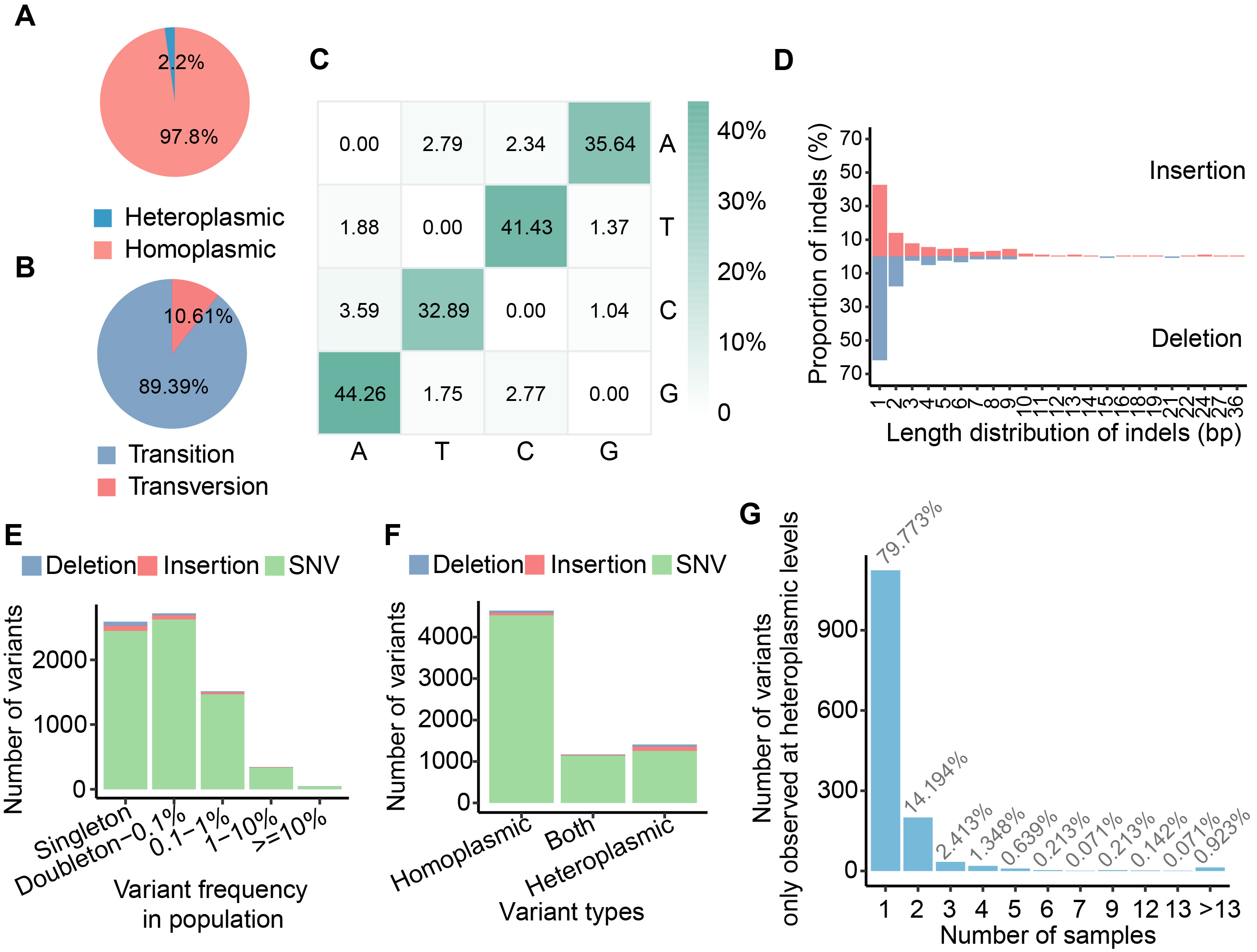

Supplement: qzaf098_Supplementary_Data [file qzaf098_supplementary_data.zip › Fig.S2.jpg]

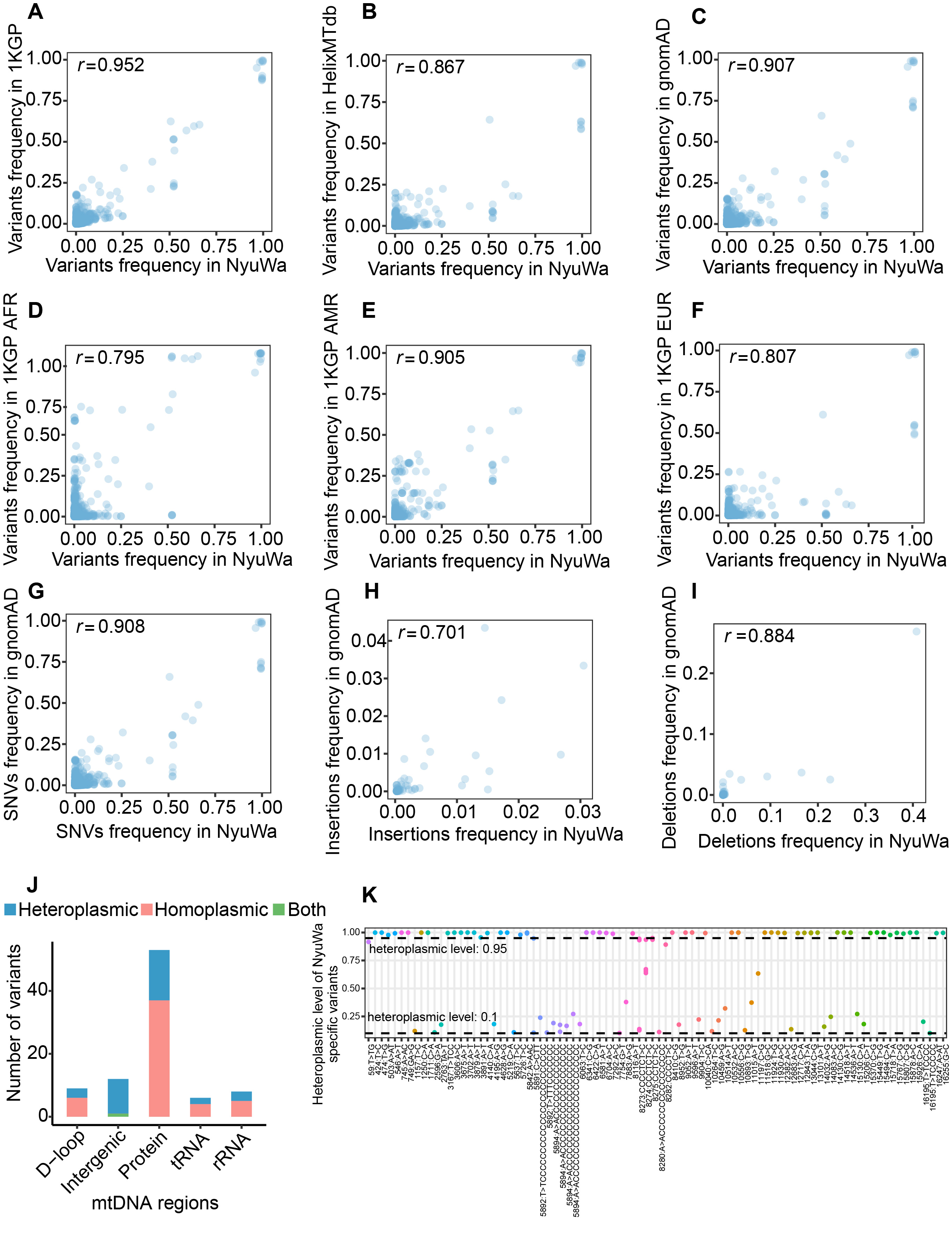

Supplement: qzaf098_Supplementary_Data [file qzaf098_supplementary_data.zip › Fig.S3.jpg]

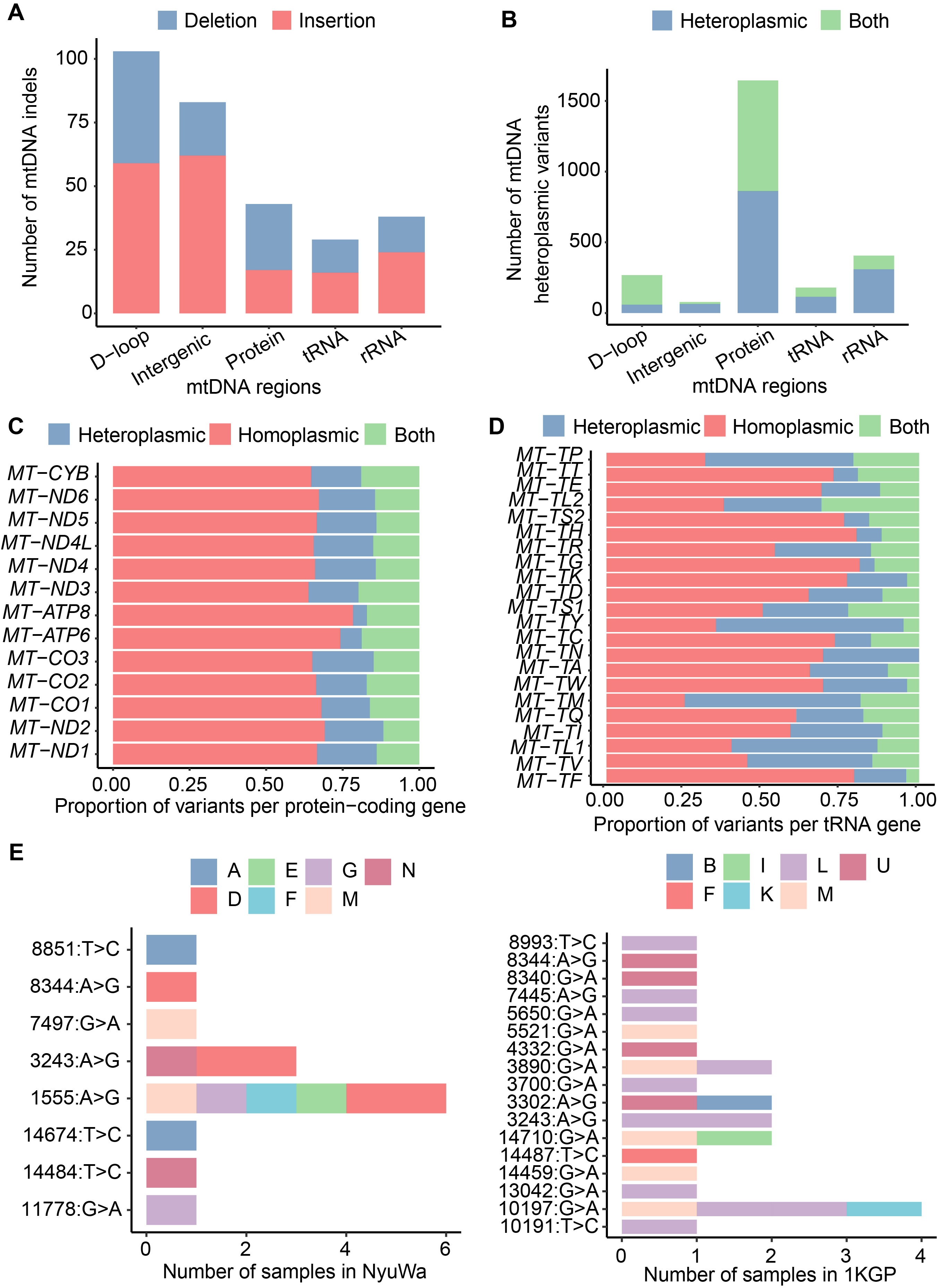

Supplement: qzaf098_Supplementary_Data [file qzaf098_supplementary_data.zip › Fig.S4.jpg]

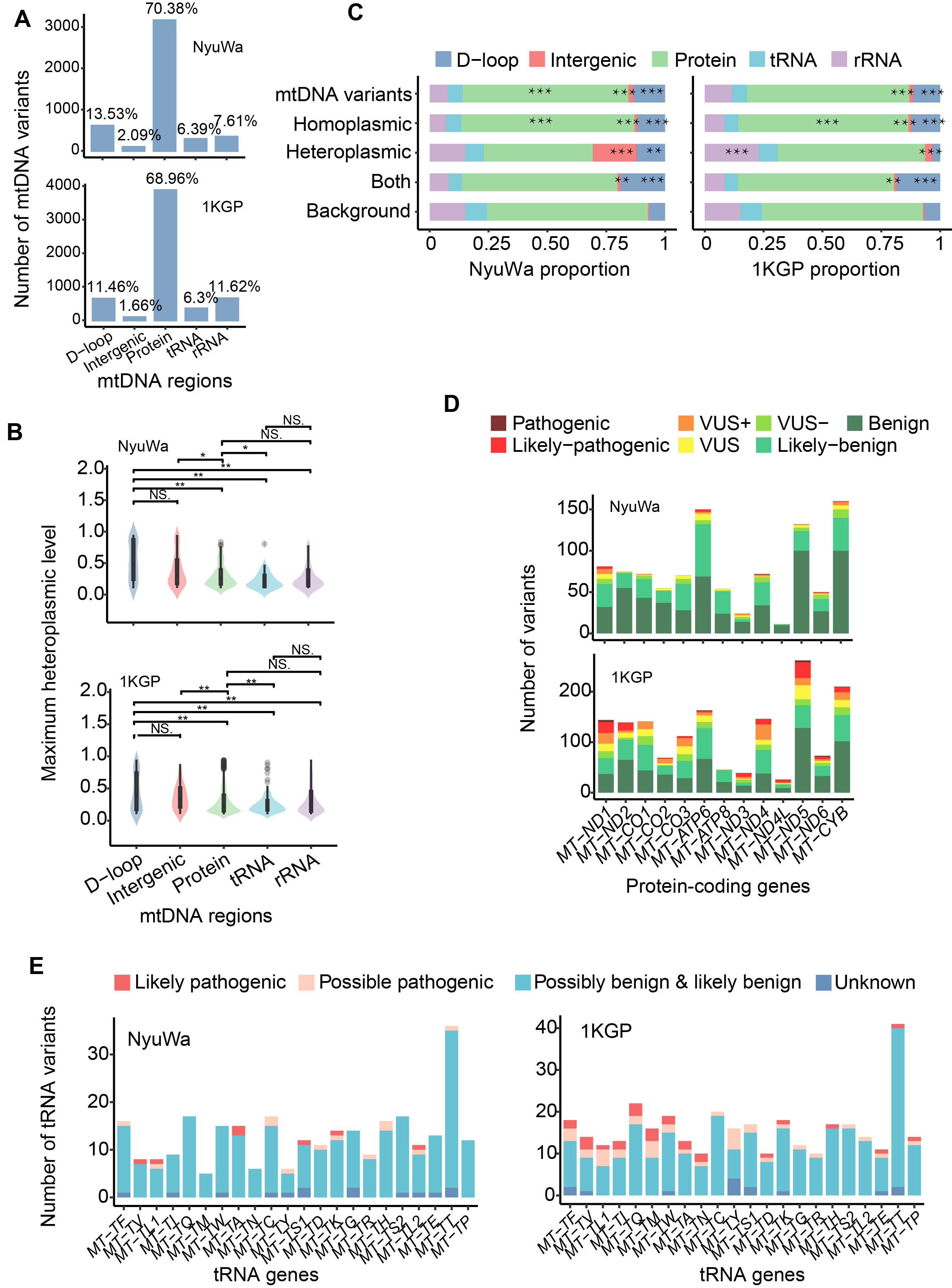

Supplement: qzaf098_Supplementary_Data [file qzaf098_supplementary_data.zip › Fig.S5.jpg]

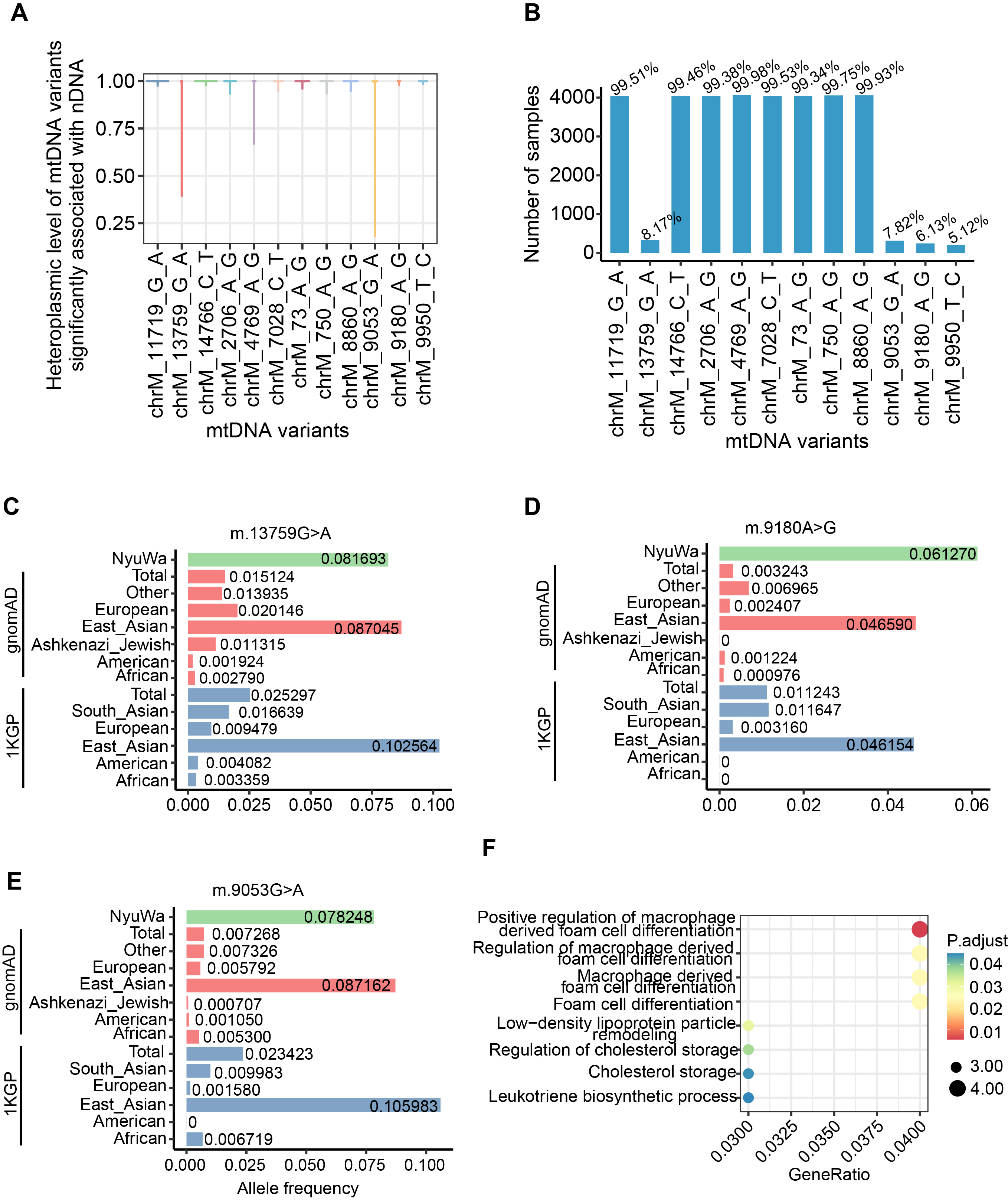

Supplement: qzaf098_Supplementary_Data [file qzaf098_supplementary_data.zip › Fig.S6.jpg]

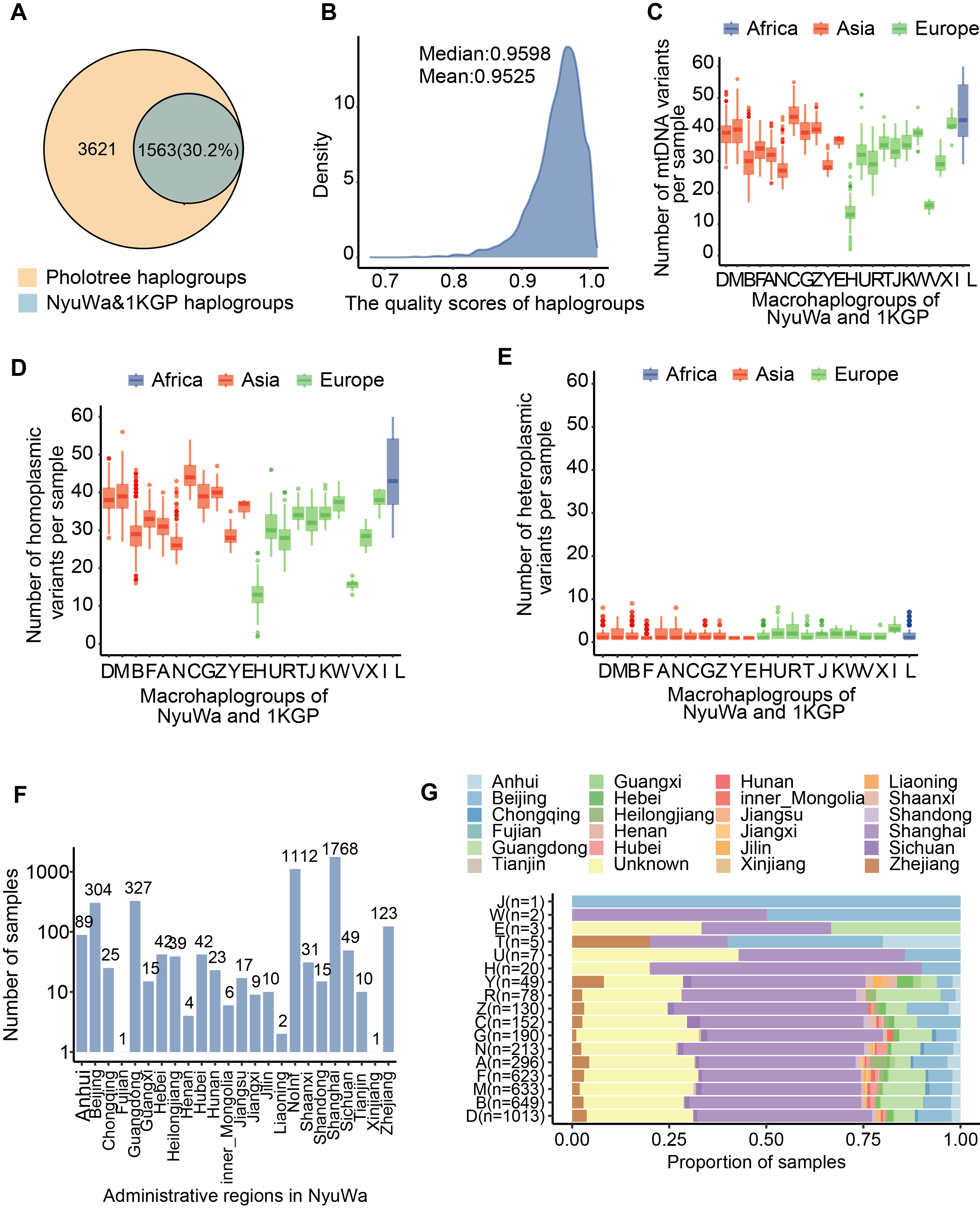

Supplement: qzaf098_Supplementary_Data [file qzaf098_supplementary_data.zip › Fig.S7.jpg]

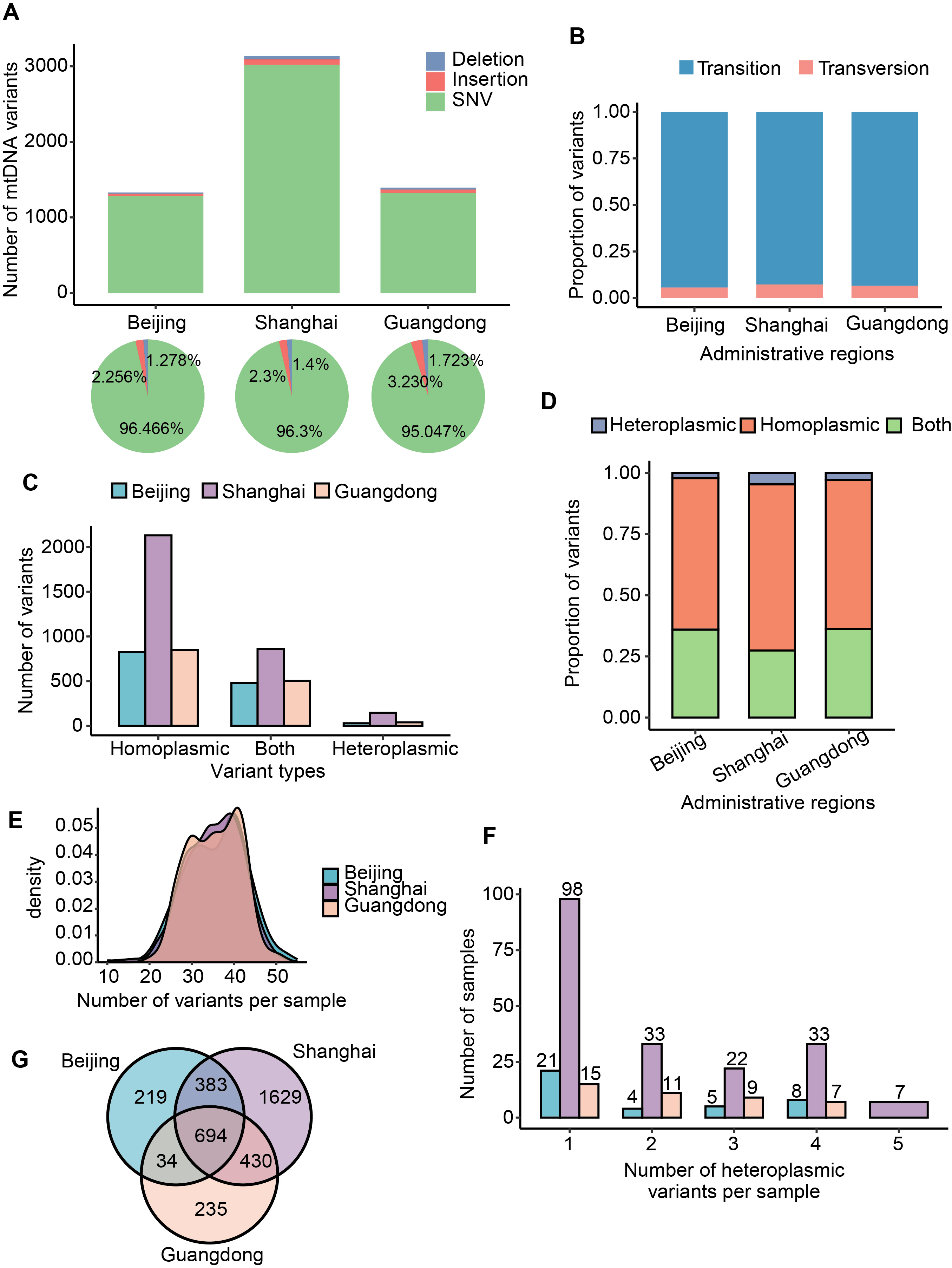

Supplement: qzaf098_Supplementary_Data [file qzaf098_supplementary_data.zip › Fig.S8.jpg]

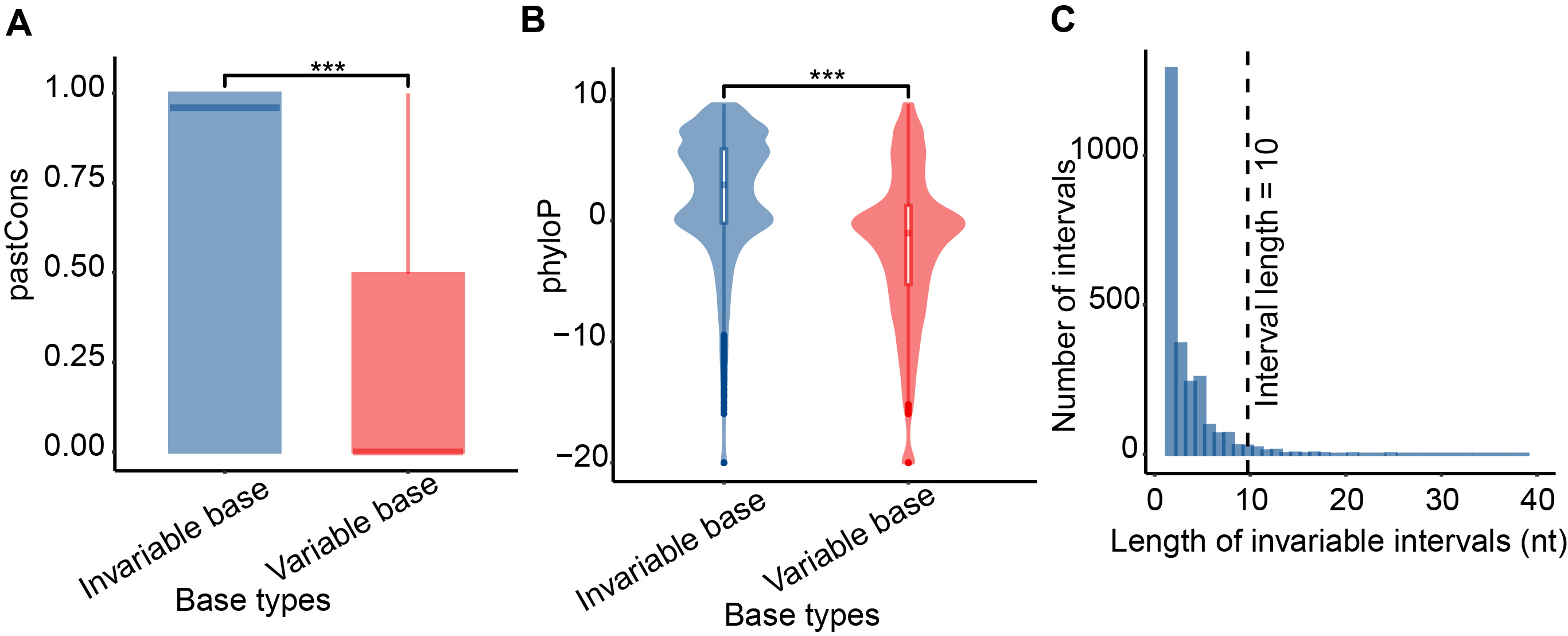

Supplement: qzaf098_Supplementary_Data [file qzaf098_supplementary_data.zip › Fig.S9.jpg]
